# Impact of Bystander Cardiopulmonary Resuscitation on Out-of-Hospital Cardiac Arrest Outcome in Vietnam

**DOI:** 10.5811/westjem.18413

**Published:** 2024-06-14

**Authors:** Co Xuan Dao, Chinh Quoc Luong, Toshie Manabe, My Ha Nguyen, Dung Thi Pham, Tra Thanh Ton, Quoc Trong Ai Hoang, Tuan Anh Nguyen, Anh Dat Nguyen, Bryan Francis McNally, Marcus Eng Hock Ong, Son Ngoc Do, The Local PAROS Investigators Group

**Affiliations:** * Bach Mai Hospital, Center for Critical Care Medicine, Hanoi, Vietnam; † Hanoi Medical University, Department of Emergency and Critical Care Medicine, Hanoi, Vietnam; ‡ Vietnam National University, University of Medicine and Pharmacy, Department of Emergency and Critical Care Medicine, Hanoi, Vietnam; § Bach Mai Hospital, Center for Emergency Medicine, Hanoi, Vietnam; ∥ Nagoya City University Graduate School of Medicine, Department of Medical Innovation, Nagoya, Aichi, Japan; ¶ Nagoya City University West Medical Center, Center for Clinical Research, Nagoya, Aichi, Japan; # Thai Binh University of Medicine and Pharmacy, Department of Health Organization and Management, Thai Binh, Vietnam; ** Thai Binh University of Medicine and Pharmacy, Department of Nutrition and Food Safety, Thai Binh, Vietnam; †† Cho Ray Hospital, Emergency Department, Ho Chi Minh City, Vietnam; ‡‡ Hue Central General Hospital, Emergency Department, Hue City, Thua Thien Hue, Vietnam; §§ Emory University School of Medicine, Department of Emergency Medicine, Atlanta, Georgia; ∥∥ Emory University Rollins School of Public Health, Atlanta, Georgia; ¶¶ Singapore General Hospital, Department of Emergency Medicine, Singapore, Singapore; ## Duke-NUS Medical School, Health Services and Systems Research, Singapore, Singapore; *** Names and affiliations listed in supplemental file

## Abstract

**Introduction:**

Patients experiencing an out-of-hospital cardiac arrest (OHCA) frequently do not receive bystander cardiopulmonary resuscitation (CPR), especially in low- and middle-income countries (LMIC). In this study we sought to determine the prevalence of OHCA patients in Vietnam who received bystander CPR and its effects on survival outcomes.

**Methods:**

We performed a multicenter, retrospective observational study of patients (≥18 years) presenting with OHCA at three major hospitals in an LMIC from February 2014–December 2018. We collected data on the hospital and patient characteristics, the cardiac arrest events, the emergency medical services (EMS) system, the therapy methods, and the outcomes and compared these data, before and after pairwise 1:1 propensity score matching, between patients who received bystander CPR and those who did not. Upon admission, we assessed factors associated with good neurological survival at hospital discharge in univariable and multivariable logistic models.

**Results:**

Of 521 patients, 388 (74.5%) were men, and the mean age was 56.7 years (SD 17.3). Although most cardiac arrests (68.7%, 358/521) occurred at home and 78.8% (410/520) were witnessed, a low proportion (22.1%, 115/521) of these patients received bystander CPR. Only half of the patients were brought by EMS (8.1%, 42/521) or private ambulance (42.8%, 223/521), 50.8% (133/262) of whom had resuscitation attempts. Before matching, there was a significant difference in good neurological survival between patients who received bystander CPR (12.2%, 14/115) and patients who did not (4.7%, 19/406; *P* < .001). After matching, good neurological survival was absent in all OHCA patients who did not receive CPR from a bystander. The multivariable analysis showed that bystander CPR (adjusted odds ratio: 3.624; 95% confidence interval 1.629–8.063) was an independent predictor of good neurological survival.

**Conclusion:**

In our study, only 22.1% of total OHCA patients received bystander CPR, which contributed significantly to a low rate of good neurological survival in Vietnam. To improve the chances of survival with good neurological functions of OHCA patients, more people should be trained to perform bystander CPR and teach others as well. A standard program for emergency first-aid training is necessary for this purpose.

Population Health Research Capsule What do we already know about this issue?

*Global survival rates for out-of-hospital cardiac arrest (OHCA) vary considerably due to differences in patients and EMS systems.*

What was the research question?

*How does the current state of bystander cardiopulmonary resuscitation (CPR) impact outcomes of OHCA in Vietnam?*

What was the major finding of the study?

*A low rate of bystander CPR (22.1%) contributed to low survival. However, bystander CPR was associated with good neurological survival (adjusted OR 3.624; 95% CI 1.629–8.063).*

How does this improve population health?

*Training more people to perform CPR and encouraging them to teach others can improve the chances of OHCA patients surviving with good neurological outcomes.*

## INTRODUCTION

Out-of-hospital cardiac arrest (OHCA) is a prominent cause of death and disability worldwide,^1^^–^^4^ accounting for up to 10% of overall mortality in low- and middle-income countries (LMIC).^5^^–^^7^ It is defined as the loss of functional cardiac mechanical activity in association with an absence of systemic circulation, occurring outside a hospital setting.^8^^,^^9^ The exact burden of OHCA on public health globally is unknown since many cases are not attended by emergency medical services (EMS), and there are often wide variations among different regions, countries, and continents in both their reporting systems and survival outcomes.^5^^,^^10^^–^^13^

In Asia-Pacific countries, EMS systems are often underdeveloped and vary considerably. Survival outcomes for OHCA in Pan-Asia differ considerably, and these variations may be related to differences in patients and the EMS system.^12^ These differences suggest that survival outcomes for OHCA can be improved by interventions to enhance EMS systems,^14^ such as increasing bystander cardiopulmonary resuscitation (CPR) through community-based CPR training programs,^15^ increasing availability of public access defibrillators,^16^ and improving post-resuscitation care.^17^ The OHCA patients in LMICs are considerably less likely to receive bystander CPR than those in high-income countries (HIC).^12^ Furthermore, in areas with underdeveloped EMS infrastructures, extremely ill or injured patients are frequently transported to hospitals by non-EMS vehicles.^18^^–^^21^

Vietnam is an LMIC with a population of 96.462 million people, ranking 15^th^ in the world and third in Southeast Asia, and it still struggles with a lack of development in prehospital services.^18^^,^^19^^,^^22^^,^^23^ The Vietnamese government implemented a countrywide strategy for the EMS system in 2008; nonetheless, only a few localities, such as urban areas, have a working EMS system. In addition, the availability of ambulances, qualified and authorized medical personnel, and life-saving equipment is restricted. Medical control and frequent monitoring of quality indicators are also uncommon.^22^ Prehospital treatment is typically left to bystanders, and the injured or sick individual is usually taken immediately to the next vehicle large enough to handle him or her; bystander CPR is also frequently not performed.^18^^–^^20^ As a result, these issues prevent the integration of prehospital and hospital treatment protocols and clinical data collection for surveillance, quality improvement, and research-related activities, and patients with life-threatening diseases or injuries are frequently not offered Basic Life Support (BLS) and Advanced Life Support (ALS) services until they arrive at the hospital.^18^^–^^20^^,^^24^

Understanding the present state of bystander CPR and how it affects the outcomes of OHCA patients locally is critical for increasing survival in Vietnam and other countries where clinical practice is hampered by inadequate medical resources. In this study we aimed to investigate the survival rates from OHCA and to compare the survival rates of non-matched and matched OHCA cohorts who received bystander CPR and who did not receive bystander CPR.

## METHODS

### Study Design and Setting

This multicenter, retrospective observational study is part of the Pan-Asian Resuscitation Outcomes Study (PAROS) Clinical Research Network, which collects data on OHCA patients admitted to hospital emergency departments (ED) in countries across Asia.^18^^,^^19^^,^^25^^,^^26^ In this study, we retrieved data from Vietnam in the PAROS database. The hospitals in Vietnam participating in the PAROS study are three public-sector, tertiary hospitals in the three largest cities of the country: Hanoi (northern Vietnam) which serves an estimated 10 million people; Hue (central Vietnam) which serves 1.154 million people; and Ho Chi Minh City (southern Vietnam) which serves 13 million people. The hospitals receive patients from all parts of each city. The reasons for selecting these institutions were as follows: 1) they are academic hospitals, responsible for educating hospital staff, treating patients who need procedures such as cardiac catheterization that cannot be performed in local hospital settings, and receiving most of the cases attended by the EMS; and 2) these three hospitals serve a diverse population of varying socioeconomic status and ethnicity. This hospital-based sample represents the general urban population in Vietnam.

Several ambulance services are available in Vietnam, but only one emergency service has an emergency number (telephone number 115), trained and accredited medical staff, life-saving equipment, medical oversight, and quality indicators that are regularly monitored.^22^^,^^27^ Several other private organizations also provide emergency services with the ability to deliver CPR, life-saving drugs, and defibrillation, or at least have a health professional trained to deal with emergencies.^28^ However, the ambulance dispatched by these organizations is not coordinated by an EMS dispatch center.^29^ For this study, we categorized the type of prehospital transportation into two groups: EMS, which refers to ambulances dispatched by an EMS dispatch center; and non-EMS, which refers to private ambulances, private transport, or public transport. We defined a private ambulance as an ambulance that was not dispatched by an EMS dispatch center. Private transport includes transport in vehicles by family members, relatives, neighbors, or passersby. Public transport includes taxis, buses, or other types of public transport.

### Participants

This study included all patients >18 years presenting with OHCA to the emergency departments (ED) of the three hospitals. We excluded OHCA patients who had suffered traumatic injury. We defined a case of OHCA as a person who was unresponsive, not breathing, and without a pulse outside the hospital setting.^30^^–^^32^ The diagnosis of OHCA or the return of spontaneous circulation (ROSC) was confirmed by EMS personnel on the scene/enroute, or by a physician in the ED. We excluded patients for whom resuscitation was not attempted by EMS or private ambulance personnel at the scene/enroute and who were immediately pronounced dead (because of rigor mortis, lividity, or “do not resuscitate” orders) at the ED. However, we included patients on whom resuscitation was attempted but who were later pronounced dead before they reached the hospital.

### Data Collection

We used a standardized classification and case record form to collect data on common variables. The data dictionary of the PAROS study is available as an online supplement to previously published papers.^12^^,^^18^ The data was extracted from emergency dispatch records, ambulance patient case notes, and ED and in-hospital records and entered into the PAROS study database using an electronic data capture system. Patient identifiers were not entered in the database to protect patient confidentiality. We then extracted data from Vietnam and merged the data sets for the three hospitals. Each hospital contributed five years of data from February 2014–December 2018.

### Variables

We included variables based on Utstein recommendations,^33^^,^^34^ such as information on the following: 1) bystander CPR; 2) availability of public access defibrillators; 3) response times; 4) provision of ALS (eg, intravenous drugs, advanced airway management including endotracheal intubation or alternative airway devices); 5) cause of the arrest (a cardiac arrest was presumed unless it was known or likely that the arrest had a non-cardiac cause (eg, asthma, terminal illness, cerebrovascular accident, drug overdose, suicide, drowning, or trauma); and 6) provision of specialized post-resuscitation care (hypothermia or extracorporeal membrane oxygenation [ECMO]). We also collected data on the location of the OHCA (eg, home, public area). We collected data on system variables; the list of variables is available as an online supplement to previously published papers.^12^^,^^18^

### Outcomes

The primary outcome of the present study was good neurological survival on hospital discharge or at day 30 post-arrest. We used the Cerebral Performance Category (CPC) score to evaluate the neurological function of the OHCA patients.^35^^,^^36^ The CPC score was calculated based on data collected from clinical records, and telephone and face-to-face interviews. In this study we defined good neurological function as a CPC score of 1 or 2,^12^ which indicates survival with mild or moderate disability. We also examined secondary outcomes that included the following: the proportions of patients in whom spontaneous circulation returned at the scene/enroute; patients who survived to hospital admission; and patients who were discharged from the hospital.

### Statistical Analyses

#### Description and Comparison of Cohorts

We report data as numbers and percentages (%) for categorical variables and medians and interquartile ranges (IQR 25–75%) or means and SDs for continuous variables. We compared OHCA patients who received bystander CPR with those who did not receive bystander CPR for each variable. We used the chi-squared test or Fisher exact test for categorical variables and the independent samples *t*-test, Mann-Whitney U test, or one-way analysis of variance for continuous variables in comparisons of these variables.

#### Matching Method

We carried out pairwise 1:1 propensity score matching (Supplementary Figure), using the nearest neighbor matching method to reduce the effect of bias by unbalanced covariates and potential confounding.^37^^,^^38^ The propensity score was estimated using multiple logistic regression analysis that included the independent variables of age (either <60 years or ≥60 years), gender (either male or female), past medical history (none, heart diseases only, other diseases, such as diabetes, cancer, hypertension, renal disease, respiratory disease, hyperlipidemia, stroke, HIV, and others, or both heart diseases and other diseases), and etiology of OHCA (either presumed cardiac or non-cardiac, such as respiratory, drowning, electrocution, and others) with bystander CPR and without bystander CPR.

#### Assessing Factors Associated with Survivability

Upon admission, we assessed factors associated with good neurological survival on hospital discharge using logistic regression analysis. To reduce the number of predictors, multicollinearity and overfitting, we used different ways to select variables. First, we started variable selection with a univariable logistic regression analysis of each variable that included independent variables related to participating hospitals, patient-related factors, cardiac arrest event-related factors, EMS system- and therapy-related factors. We included variables for consideration in the multivariable logistic regression analysis if the *P*-value was <0.05 in the univariable logistic regression analysis, as well as factors that were clinically important (including age, past medical history, presence of a witness, etiology of OHCA, type of prehospital transportation and bystander CPR). Second, we used a stepwise backward elimination method to select variables for multivariable logistic regression analysis. Similarly, we used these methods of variable selection and analysis to assess factors associated with survival to hospital admission and survival to hospital discharge. We present odds ratios (OR) and 95% confidence intervals (CI).

We used SPSS Statistics 25.0 (SPSS, Inc, Chicago, IL) for data analysis. For all statistical analyses, significance levels were two-tailed, and we considered *P* < 0.05 as statistically significant.

## RESULTS

During the study period, 779 OHCA patients had their data submitted to the PAROS database. We removed from the study 31 individuals <18 years old and 109 with traumatic injuries. We additionally removed 30 patients (4.69%; 30/639) due to a prolonged prehospital stay (ie, more than one day), which might have indicated input mistakes or enrollment of patients transferred from the referring hospitals. Moreover, we excluded 88 patients (13.77%; 88/639) from our analysis due to the absence of most variables. In total we included 521 eligible patients in our analyses (Figure).

**Figure.**
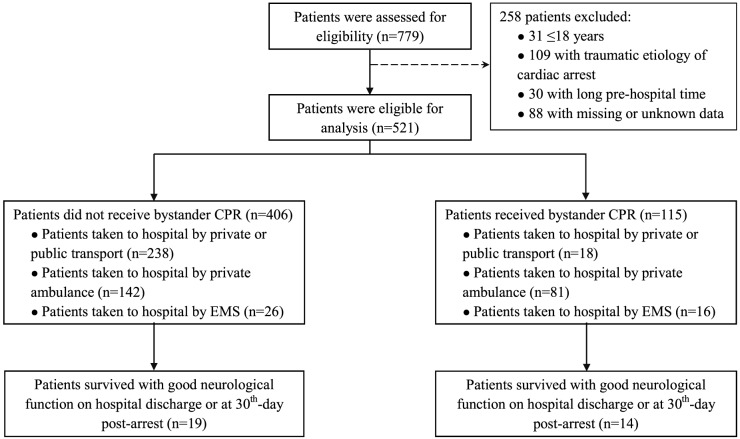
Flowchart of type of bystander cardiopulmonary resuscitation, transportation to the hospital, and outcome of patients with out-of-hospital cardiac arrest included in the study, Vietnam, February 2014–December 2018. *CPR*, cardiopulmonary resuscitation; *EMS*, emergency medical services.

### The Primary and Secondary Outcomes

Of the 521 OHCA patients, 98 (18.8%) had a ROSC at the scene/enroute, and for 113 (21.7%) patients, spontaneous circulation returned in the ED (Table 1). Overall, 18.4% (96/521) of patients survived on hospital admission, and 9.4% (49/521) survived to hospital discharge; 6.3% (33/521) survived with good neurological function (a CPC score of 1 or 2) on hospital discharge or at 30th-day post-arrest (Table 1).

**Table 1.** Outcomes of non-matched and matched cohorts of patients with out-of-hospital cardiac arrest according to the type of bystander cardiopulmonary resuscitation, Vietnam, February 2014–December 2018.

Variables	Before matching				After matching			
	All cases (n = 521)	No bystander CPR (n = 406)	Bystander CPR (n = 115)	*P*-value^a^	All cases (n = 212)	No bystander CPR (n = 106)	Bystander CPR (n = 106)	*P*-value^b^
ROSC, no. (%)								
ROSC at scene/enroute	98 (18.8)	57 (14.0)	41 (35.7)	<0.001	39 (18.4)	0 (0.0)	39 (36.8)	<0.001
ROSC at ED	113 (21.7)	81 (20.00	32 (27.8)	0.07	49 (23.1)	18 (17.0)	31 (29.2)	0.03
Outcome of patient at ED, no. (%)				0.06				<0.001
Died in ED	425 (81.6)	338 (83.3)	87 (75.7)		185 (87.3)	106 (100.0)	79 (74.5)	
Admitted	96 (18.4)	68 (16.7)	28 (24.3)		27 (12.7)	0 (0.0)	27 (12.7)	
Patient status, no. (%)				0.14				<0.001
Died in the hospital	41 (7.9)	31 (7.6)	10 (8.7)		10 (4.7)	0 (0.0)	10 (9.4)	
Remains in hospital at day 30 post arrest	6 (1.2)	5 (1.2)	1 (0.9)		0 (0.0)	0 (0.0)	0 (0.0)	
Discharged alive	49 (9.4)	32 (7.90	17 (14.8)		17 (8.0)	0 (0.0)	17 (16.0)	
Post arrest CPC 1 or 2, n (%)	33 (6.3)	19 (4.7)	14 (12.2)	<.001	14 (6.6)	0 (0.0)	14 (13.2)	<0.001

a The comparison between patients who did not receive bystander CPR and who received bystander CPR in the non-matched cohort.

b The comparison between patients who did not receive bystander CPR and who received bystander CPR in the matched cohort.

*CPC*, cerebral performance category; *CPR*, cardiopulmonary resuscitation; *ED*, emergency department; *ROSC*, return of spontaneous circulation.

### Clinical Characteristics and Pre-Hospital and In-Hospital Management

Among the total number of OHCA patients, 74.5% (388/521) were men and the mean age was 56.7 years (SD 17.3). Less than a fifth of the patients (18.1%; 85/470) had a past medical history of heart disease (Table 2). Most OHCAs occurred at home (68.7%; 358/521) and during the day (56.6%; 181/320) (Table 3). The witnessed OHCAs accounted for 78.8% (410/520) of patients (Table 3), most of which were bystander-witnessed cardiac arrests, including layperson (4.2%; 22/520), family members (13.8%; 72/520), and healthcare professionals (49.8%; 259/520). A cardiac condition was the presumed cause of cardiac arrest in 44.9% (234/521) of patients (Table 3). Of the 521 OHCA patients, 49.1% (256/521) were taken to hospital by private or public transport, 42.8% (223/521) were taken by private ambulance, and only 8.1% (42/521) were taken by EMS (Table 4 and Supplementary Table 1).

**Table 2.** Patient-related characteristics of non-matched and matched cohorts of patients with out-of-hospital cardiac arrest according to the type of bystander cardiopulmonary resuscitation, Vietnam, February 2014–December 2018.

Variables	Before matching				After matching			
	All cases (n = 521)	No bystander CPR (n = 406)	Bystander CPR (n = 115)	*P*-value^a^	All cases (n = 212)	No bystander CPR (n = 106)	Bystander CPR (n = 106)	*P*-value^b^
Hospital participated								
Hospital				0.03				0.14
Bach Mai, no. (%)	396 (76.0)	306 (75.4)	90 (78.3)		176 (83.0)	91 (85.8)	85 (80.2)	
Hue, no. (%)	24 (4.6)	24 (5.9)	0 (0.0)		2 (0.9)	2 (1.9)	0 (0.0)	
Cho Ray, no. (%)	101 (19.4)	76 (18.7)	25 (21.7)		34 (16.0)	13 (12.3)	21 (19.8)	
Patient-related								
Age (year), mean (SD)	56.7 (17.3)	57.6 (17.2)	53.7 (17.6)	0.04	56,6 (17.5)	60.0 (16.6)	53.1 (17.8)	<.001
Gender (male), no. (%)	388 (74.5)	305 (75.10	83 (72.2)	0.52	154 (72.6)	78 (73.6)	76 (71.7)	0.76
Past medical history, no. (%), n1 = 470^c^								
Heart disease	85 (18.1)	60 (16.5)	25 (23.6)	0.10	38 (17.9)	13 (12.3)	25 (23.6)	0.03
Diabetes	64 (13.6)	46 (12.6)	18 (17.00	0.30	30 (14.2)	12 (11.3)	18 (17.0)	0.24
Cancer	38 (8.1)	34 (9.3)	4 (3.8)	0.06	11 (5.2)	7 (6.6)	4 (3.8)	0.35
Hypertension	111(23.6)	85 (23.4)	26 (24.5)	0.80	47 (22.2)	21 (19.8)	26 (24.5)	0.41
Renal disease	38 (8.1)	27 (7.4)	11 (10.4)	0.33	15 (7.1)	4 (3.8)	11 (10.4)	0.06
Respiratory disease	75 (16.0)	53 (14.6)	22 (20.8)	0.13	37 (17.5)	15 (14.2)	22 (20.8)	0.21
Hyperlipidemia	4 (0.9)	4 (1.1)	0 (0.0)	0.58	0 (0.0)	0 (0.0)	0 (0.0)	NA
Stroke	16 (3.4)	15 (4.1)	1 (0.9)	0.14	6 (2.8)	5 (4.7)	1 (0.9)	0.21
HIV	1 (0.2)	1 (0.3)	0 (0.0)	> 0.99	0 (0.0)	0 (0.0)	0 (0.0)	NA

a The comparison between patients who did not receive bystander CPR and who received bystander CPR in the non-matched cohort.

b The comparison between patients who did not receive bystander CPR and who received bystander CPR in the matched cohort.

c n1 was defined as the total number of patients recorded if a variable was given or not in the non-matched cohort.

*CPR*, cardiopulmonary resuscitation; *NA*, not available.

**Table 3.** Event-related characteristics of non-matched and matched cohorts of patients with out-of-hospital cardiac arrest according to the type of bystander cardiopulmonary resuscitation, Vietnam, February 2014–December 2018.

Variables	Before matching				After matching			
	All cases (n = 521)	No bystander CPR (n = 406)	Bystander CPR (n = 115)	*P*-value^a^	All cases (n = 212)	No bystander CPR (n = 106)	Bystander CPR (n = 106)	*P*-value^b^
Location type, n (%)				<0.001				<0.001
In EMS/private ambulance	63 (12.1)	46 (11.3)	17 (14.8)		40 (18.9)	24 (22.6)	16 (15.1)	
Healthcare facility	50 (9.6)	14 (3.4)	36 (31.3)		40 (18.9)	8 (7.5)	32 (30.2)	
Home residence	358 (68.7)	304 (74.9)	54 (47.0)		109 (51.4)	59 (55.7)	50 (47.2)	
Public area	50 (9.6	42 (10.3	8 (7.0		23 (10.8)	15 (14.2)	8 (7.5)	
Time of the day, no. (%), n1 = 320^d^, n2 = 105^d^	181 (56.6)	125 (53.0)	56 (66.7)	0.03	64 (61.0)	13 (44.8)	51 (67.1)	0.04
Witnessed arrest, n1 = 520^d^	410 (78.8)	297 (73.30	113 (98.3)	<0.001	128 (60.4)	24 (22.6)	104 (98.1)	<0.001
Arrest witnessed by, no. (%), n1 = 520^d^				<0.001				<0.001
Not witnessed	110 (21.2)	108 (26.7)	2 (1.7)		84 (39.6)	82 (77.4)	2 (1.9)	
Bystander (lay person)	22 (4.2)	16 (4.0)	6 (5.2)		11 (5.2)	6 (5.7)	5 (4.7)	
Bystander (family)	72 (13.8)	19 (4.7)	53 (46.1)		65 (30.7)	16 (15.1)	49 (46.2)	
Bystander (healthcare worker)	259 (49.8)	229 (56.5)	30 (26.1)		31 (14.6)	2 (1.9)	29 (27.4)	
EMS/private ambulance	57 (11.0)	33 (8.1)	24 (20.9)		21 (9.9)	0 (0.0)	21 (19.8)	
Presumed cardiac etiology of OHCA	234 (44.9)	184 (45.3)	50 (43.5)	0.73	82 (38.7)	36 (34.0)	46 (43.4)	0.16
Shockable first arrest rhythms^c^, n1 = 135^d^, n2 = 56^d^	93 (68.9)	51 (67.1)	42 (71.2)	0.61	39 (69.6)	NA	39 (69.6)	NA

a The comparison between patients who did not receive bystander CPR and who received bystander CPR in the non-matched cohort.

b The comparison between patients who did not receive bystander CPR and who received bystander CPR in the matched cohort.

c Shockable first arrest rhythms included ventricular tachycardia, ventricular fibrillation, or unknown shockable rhythms.

d n1 and n2 were defined as the total number of patients recorded if a variable was given or not in the non-matched and matched cohorts.

*CPR*, cardiopulmonary resuscitation; *EMS*, emergency medical services; *NA*, not available; *OHCA,* out-of-hospital cardiac arrest.

**Table 4.** System-related characteristics of non-matched and matched cohorts of patients with out-of-hospital cardiac arrest according to the type of bystander cardiopulmonary resuscitation, Vietnam, February 2014–December 2018.

Variables	Before matching				After matching			
	All cases (n = 521)	No bystander CPR (n = 406)	Bystander CPR (n = 115)	*P*-value^a^	All cases (n = 212)	No bystander CPR (n = 106)	Bystander CPR (n = 106)	*P*-value^b^
Prehospital transport, no. (%)				<0.001				<0.001
EMS	42 (8.1)	26 (6.4)	16 (13.9)		16 (7.5)	1 (0.9)	15 (14.2)	
Private ambulance	223 (42.8)	142 (35.0)	81 (70.4)		111 (52.4)	37 (34.9)	74 (69.8)	
Private or public transport	256 (49.1)	238 (58.6)	18 (15.7)		85 (40.1)	68 (64.2)	17 (16.0)	
Resuscitation attempted by EMS/private ambulance, no. (%), n1 = 262^c^, n2 = 125^c^	133 (50.8)	75 (44.6)	58 (61.7)	0.01	55 (44.0)	0 (0.0)	55 (63.2)	<0.001
Time to initiation of CPR (min), mean (SD), n1 = 87^c^	7.3 (8.7)	9.1 (5.6)	5.1 (11.1)	<0.001	5,5 (11.5)	NA	5,5 (11.5)	NA
Time to defibrillation at scene (min), mean (SD), n2 = 36^c^	9.0 (6.2)	9.7 (5.1)	7.7 (7.9)	0.13	8.5 (8.4)	NA	8.5 (8.4)	NA

a The comparison between patients who did not receive bystander CPR and who received bystander CPR in the non-matched cohort.

b The comparison between patients who did not receive bystander CPR and who received bystander CPR in the matched cohort.

c n1 and n2 were defined as the total number of patients recorded if a variable was given or not in the non-matched and matched cohorts.

*CPR*, cardiopulmonary resuscitation; *EMS*, emergency medical services; *NA*, not available.

Only 31.9% (43/135) of OHCA patients received prehospital defibrillation (Table 5). Only 22.1% (115/521) of the patients received bystander CPR, and 5.3% (14/262) received a bystander automated external defibrillator (AED) (Table 5). Epinephrine was given to 23.4% (122/521) of patients with cardiac arrest at the scene/enroute, and 20.7% (108/521) received prehospital advanced airway management (Table 5). Hypothermia therapy was given to 15.0% (78/521) of OHCA patients, but only 1.3% (7/519) were given ECMO therapy (Table 5). The characteristics, management, and complications of the study cohort are shown in Tables 2, 3, 4, and 5.

**Table 5.** Therapy-related characteristics of non-matched and matched cohorts of patients with out-of-hospital cardiac arrest according to the type of bystander cardiopulmonary resuscitation, Vietnam, February 2014–December 2018.

Variables	Before matching				After matching			
	All cases (n = 521)	No bystander CPR (n = 406)	Bystander CPR (n = 115)	*P*-value^a^	All cases (n =212)	No bystander CPR (n = 106)	Bystander CPR (n = 106)	*P*-value^b^
Pharmacotherapy, no. (%)								
Epinephrine (at scene)	122 (23.4)	67 (16.5)	55 (47.8)	<0.001	52 (24.5)	0 (0.0)	52 (49.1)	<0.001
Epinephrine (at ED)	480 (92.1)	374 (92.1)	106 (92.2)	>0.99	196 (92.5)	99 (93.4)	97 (91.5)	0.60
Prehospital intervention, no. (%)								
Prehospital defibrillation, n1 = 135^c^, n2 = 56^c^	43 (31.9)	29 (38.2)	14 (23.7)	0.07	12 (21.4)	NA	12 (21.4)	NA
Bystander AED applied, n1 = 262^c^, n2 = 125^c^	14 (5.3)	7 (4.2)	7 (7.4)	0.26	7 (5.6)	0 (0.0)	7 (8.0)	0.10
ED defibrillation performed, no. (%)	68 (13.1)	48 (11.8)	20 (17.4)	0.12	24 (11.3)	6 (5.7)	18 (17.0)	0.01
Prehospital advanced airway, no. (%)	108 (20.7)	62 (15.3)	46 (40.0)	<0.001	43 (20.3)	0 (0.0)	43 (40.6)	<0.001
Advanced airway used at ED, no. (%)	297 (57.0)	241 (59.4)	56 (48.7)	0.04	111 (52.4)	59 (55.7)	52 (49.1)	0.34
Admission coronary angiography, no. (%)								
Emergency PCI performed	23 (4.4)	18 (4.4)	5 (4.3)	0.97	5 (2.4)	0 (0.0)	5 (4.7)	0.06
Emergency CABG performed	2 (0.4)	2 (0.5)	0 (0.0)	>0.99	NA	NA	NA	NA
Post-resuscitation care, no. (%)								
ECMO therapy initiated, n1 = 519^c^	7 (1.30)	5 (1.2)	2 (1.7)	0.65	2 (0.9)	1 (0.9)	1 (0.9)	>0.99
Hypothermia therapy initiated	78 (15.0)	53 (13.1)	25 (21.7)	0.02	26 (12.3)	2 (1.9)	24 (22.6)	<0.001

a The comparison between patients who did not receive bystander CPR and who received bystander CPR in the non-matched cohort.

b The comparison between patients who did not receive bystander CPR and who received bystander CPR in the matched cohort.

c n1 and n2 were defined as the total number of patients recorded if a variable was given or not in the non-matched and matched cohorts.

*AED*, automated external defibrillation; *CABG*, coronary artery bypass grafting; *CPR*, cardiopulmonary resuscitation; *ECMO*, extracorporeal membrane oxygenation therapy; *ED*, emergency department; *NA*, not available; *PCI*, percutaneous coronary intervention.

### Impact of Bystander CPR on the Outcomes

In non-matched and matched cohorts, Tables 1, 2, 3, 4, and 5 compare the general characteristics, prehospital and in-hospital treatment, and outcomes of OHCA patients who did not receive bystander CPR to those who did.

#### In Non-Matched Cohort

There was a significant difference in resuscitation attempted by EMS or private ambulance between patients who received bystander CPR (61.7%; 58/94) and patients who did not receive bystander CPR (44.6%; 75/168; *P* =  0.01) (Table 4). The proportion of patients in whom spontaneous circulation returned at the scene/enroute was significantly higher in patients who received bystander CPR (35.7%; 41/115) compared to patients who did not receive bystander CPR (14.0%; 57/406; *P* < 0.001) (Table 1). However, there was no significant difference in survival to hospital admission between patients who received bystander CPR (24.3%; 28/115) and patients who did not (16.7%; 68/406; *P* = 0.06) and survival to hospital discharge between patients who received bystander CPR (14.8%; 17/115) and patients who did not (7.9%; 32/406; *P* = 0.14) (Table 1). In contrast, the rate of good neurological survival on hospital discharge or at day 30 post-arrest in patients who received bystander CPR (12.2%, 14/115) was significantly higher than that in patients who did not receive bystander CPR (4.7%, 19/406; *P* < .001) (Table 1).

#### In Matched Cohort

We used propensity score matching to obtain 106 pairs of patients with similar characteristics (Tables 1, 2, 3, 4, and 5). Among OHCA patients who did not receive bystander CPR, none received resuscitation attempted by EMS or private ambulance (Table 4) or had ROSC at the scene/enroute (Table 1). As a result, none of the OHCA patients survived on hospital admission or obviously survived to hospital discharge (Table 1).

### Association of Bystander CPR with Survivability

In contrast to the association between bystander CPR and survival to hospital admission (Supplementary Table 2), Supplementary Tables 3 and 4 show bystander CPR was identified in the univariable logistic regression to be significantly associated with increased chance of survival to hospital discharge (OR 2.027; 95% CI 1.081–3.802) and good neurological survival on hospital discharge or at day 30 post-arrest (OR 2.823; 95% CI 1.368–5.825). However, the multivariable logistic regression showed that bystander CPR was independently associated with only an increased chance of good neurological survival on hospital discharge or at day 30 post-arrest (adjusted OR 3.624; 95% CI 1.629–8.063) (Table 6). Other factors were associated with survivability, as shown in Table 6 and Supplementary Tables 2, 3, and 4.

**Table 6.** Factors related to survival outcomes in a non-matched cohort of patients with out-of-hospital cardiac arrest in Vietnam, February 2014–December 2018: multivariable logistic regression analyses.

Factors	Survival to admission^a^		Survival to discharge^b^		Good neurological function^c^	
	AOR (95% CI)	*P*-value	AOR (95% CI)	*P*-value	AOR (95% CI)	*P*-value
*Patient-related*						
Age ≥ 60 years	0.545 (0.311–0.955)	0.03	0.329 (0.155–0.698)	<0.001	0.273 (0.106–0.702)	0.01
Past medical history						
Heart diseases	NA	NA	0.073 (0.015–0.356)	<0.001	0.027 (0.003–0.265)	<0.001
Cancer	0.167 (0.038–0.740)	0.02	NA	NA	NA	NA
Renal disease	0.059 (0.008–0.453)	0.01	NA	NA	NA	NA
Respiratory disease	2.490 (1.320–4.697)	0.01	4.310 (1.869–9.941)	<0.001	8.386 (2.834–24.812)	<0.001
*Event-related*						
Location type						
In EMS/private ambulance	Reference	<0.001	NA	NA	NA	NA
Healthcare facility	3.175 (0.679–14.848)	0.14	NA	NA	NA	NA
Home residence	7.827 (2.294–26.708)	<0.001	NA	NA	NA	NA
Public area	10.330 (2.384–44.757)	<0.001	NA	NA	NA	NA
Witnessed arrest	3.657 (1.471–9.091)	0.01	3.625 (1.057–12.431)	0.04	NA	NA
Presumed cardiac etiology	NA	NA	3.337 (1.570–7.094)	<0.001	7.236 (2.611–20.053)	<0.001
*System-related*						
Prehospital transportation						
Private or public transport	0.204 (0.106–0.392)	<0.001	NA	NA	NA	NA
*Therapy-related*						
Bystander CPR	NA	NA	1.962 (0.980–3.929)	0.06	3.624 (1.629–8.063)	<0.001
Constant	0.024	<0.001	0.023	<0.001	0.022	<0.001

a Indicate the patient received hospital admission.

b Indicate whether the patient was discharged alive or remained in the hospital on the day 30 post-arrest.

c Indicate the patient’s neurological outcome at the time of discharge or the 30^th^ day after the cardiac arrest.

*AOR,* adjusted odds ratio; *CI*, confidence interval; *CPR*, cardiopulmonary resuscitation; *EMS*, Emergency Medical Services; *NA*, not available; *OHCA*, out-of-hospital cardiac arrest.

## DISCUSSION

Of 521 OHCA patients included in our analysis, just over one-fifth (22.1%) received bystander CPR. As a result, less than one-fifth (18.4%) of these patients survived to hospital admission, only one-tenth (9.4%) were discharged from the hospital, and just over one-twentieth (6.3%) were discharged from the hospital with good neurological function (Table 1). Our study found that the survival rate of medical OHCA patients on admission aligns with the rate (20.4%; 8,341/40,878) reported by the French national registry.^39^ This could be due to the Franco-German EMS model, where physicians often accompany patients in ambulances.^40^ However, our results surpass a previous study in Hanoi, Vietnam, which reported lower rates of bystander CPR (8.4%; 20/239), survival at discharge (3.8%; 9/239), and good neurological survival (0.4%; 1/239).^20^

The differences could be due to the distinct inclusion criteria between the studies. For instance, our study included OHCA patients who received resuscitation attempts by EMS/private ambulance personnel at the scene/enroute and excluded those with traumatic injuries. Despite having a lower rate of bystander CPR, our study has a higher rate of survival to discharge than the rate reported in a retrospective cohort study in Thailand (3.4%; 42/1240),^41^ and even has a higher rate of survival to discharge than the rates reported in studies in Japan (5.2%; 2,677/51,377), Korea (8.5%; 681/7,990), and Singapore (2.5%; 76/3,023).^12^ Our rate for good neurological survival to hospital discharge is also higher than the rates reported in these countries: Thailand (1.6%; 9/573); Japan (2.8%; 1,436/51,377); Korea (3.0%; 236/7,990); and Singapore (1.7%; 50/3,023).^12^

We recognize that our cohort is likely to be a highly selected population, as many OHCA patients in Vietnam are not brought to the hospital and die outside the hospital setting.^42^^–^^44^ These findings could be due to a selection bias in our study, as we only had data on patients brought to the three highest level public sector hospitals in Vietnam. Furthermore, we included OHCA patients brought to the hospital by EMS/private ambulances. Among these patients, there were no cases for whom resuscitation was not attempted by EMS/private ambulance personnel at the scene/enroute and then were immediately pronounced dead at the ED. These might inflate the survival rate. Therefore, these cases may not reflect all OHCAs in the country.

A pivotal component in successful resuscitation from OHCA is the chain of survival.^45^^,^^46^ Rapid public-access defibrillation (PAD) with AEDs and bystander CPR improve survival rates.^6^^,^^47^^–^^50^ However, our study found that a small number of OHCAs receiving bystander CPR still considerably influenced the lower overall survival rates (Table 1). Most patients not receiving bystander CPR were taken to the hospital by private or public transport (Table 4), usually without first-aid.^24^^,^^42^^,^^43^^,^^51^ In such situations, bystander first-aid is vital for OHCA outcomes.^52^ However, bystander CPR is rarely performed in Vietnam,^24^ which could be due to the lack of knowledge, absence of dispatcher-assisted CPR (T-CPR) programs, fear of harm or infection, and legal concerns^53^ that may prevent bystanders from using such techniques (eg, CPR, PAD) and using them effectively.^54^ Most CPR-willingness studies have been conducted in HICs,^53^^,^^55^ with few in LMICs.

A study in Lebanon discovered a negative correlation between the lack of previous training and confidence in performing CPR and the willingness to do CPR in OHCA patients.^54^ It is clear that timely CPR and defibrillation, regardless of who does them, are crucial for improving survival rates from OHCAs.^56^ While enhancing EMS response times is challenging and potentially costly, simplified training programs can engage the public effectively. For instance, a focus on compression-only CPR has increased bystander CPR rates and survival rates.^57^ The aim should not be to dilute the quality of CPR training but to extend outreach to more individuals in the community to build a pyramid of first responders.^14^ To improve bystander first-aid in Vietnam, more laypeople should be trained through a recognized emergency first-aid program.^58^ Plans for the future should include dedicated training and quality improvement activities for T-CPR at dispatch centers.

In our study, EMS attended to and transported a small number of OHCA patients to the hospital (Table 4). This finding might be attributed to a lack of resources, knowledge, and infrastructure for emergency medical treatment, such as EMS dispatch centers.^22^^,^^27^ Despite economic and political changes that have resulted in strong economic growth in Vietnam,^59^ ambulances, qualified and accredited medical personnel, and life-saving equipment are in short supply. Medical supervision and frequent monitoring of quality indicators are also rare.^22^^,^^27^ At the same time, recruiting new EMS workers or healthcare practitioners is fraught with difficulties.^27^ For example, following graduation, all doctors and nurses must complete an 18-month clinical training program in inpatient settings to obtain their complete clinical license.^28^ However, EMS is not recognized as a clinical training facility, which makes obtaining postgraduate certification difficult. As a result, ambulance medical staff are understaffed, overworked, and underequipped; and EMS centers are overburdened.^27^ Moreover, call center staff do not have the ability to identify a possible person in cardiac arrest and provide CPR instructions to callers.^22^ Public bystanders are also reluctant to call EMS, and this may explain why in our study we found that a very low proportion of OHCA patients received bystander CPR or were taken to the hospital by EMS.

In 2011, the Ministry of Health began issuing licenses for private ambulances to provide first-aid or patient transportation.^28^ These services are equipped to perform CPR, administer life-saving drugs, use defibrillators, and generally have a medical professional on board trained to handle emergencies. However, our study found that only about two-fifths of OHCA patients were transported by these services. A significant number of these patients did not receive CPR from bystanders (Table 4). Moreover, for OHCA patients who did not receive CPR from bystanders, resuscitation attempts were often not performed by EMS/private ambulance personnel (Table 4). These findings could be due to limited medical interventions provided by some private organizations and healthcare workers’ difficulty in recognizing cardiac arrests.^29^ Bystanders might also be unwilling to call private ambulance services; the injured or sick person or OHCA patient is often carried quickly by the nearest private vehicle large enough to accommodate them and brought to the hospital by friends and relatives.^24^^,^^29^^,^^42^

In this study, univariable logistic regression identified two factors as significantly lowering the likelihood of good neurological survival at hospital discharge: patients who were transported to the hospital by private or public transportation, and patients who did not receive bystander CPR (Supplementary Table 4). Comparatively, those who received bystander CPR were found in multivariable logistic regression to be independently related to a high probability of surviving until hospital discharge with good neurological function (Table 6). These findings highlight the most important factor that strongly predicted good neurological survival at hospital discharge was bystander CPR, which overwhelmed other factors included in our multivariable logistic regression (Table 6). These findings also mean that bystander CPR plays the first crucial role in the chain of survival, regardless of the type of prehospital transport.^14^^,^^45^^,^^46^^,^^57^^,^^60^

## LIMITATIONS

There are several limitations to this study. Firstly, our study was limited by its retrospective design. As a result, our data was missing many variables. For instance, we only had information on whether resuscitation attempts were made by EMS/private ambulance personnel for 262 patients. Moreover, most time-stamped data was absent for various events (eg, response times), and we excluded 88 patients from our analysis due to the absence of most variables. These limitations have resulted in an implicit selection bias, hindered our ability to calculate a higher propensity score, and limited any potential definitive conclusions. Secondl*y*, it is not feasible to ascertain whether bystander CPR adhered to the American Heart Association or Red Cross protocol. Consequently, bystander CPR may vary significantly and not align with standard recommendations.

Thirdly, our study was conducted in three of the highest level public sector hospitals in Vietnam and focused on a highly selected population of cases. However, the study did not include patients brought to the hospital by EMS/private ambulances who were pronounced dead in the field. As a result, the number of persons suffering from OHCA is expected to be much larger than what was reported in this hospital-based study. Additionally, we found that many OHCA patients arrived at the hospital by private transportation rather than EMS/private ambulances. Some of these individuals may have been seen by primary care doctors, may have died at home, or may not have been transported to the hospital at all. Moreover, the number of OHCA patients varied significantly across hospitals. This difference is because the Hue Central General and the Cho Ray Hospitals had only a small number of patients enrolled in 2017 and 2018. Thus, these factors have also resulted in an implicit selection bias or incomplete enrolment and inclusion of patients in the OHCA database. Differences in figures found between Vietnam and other countries might be accounted for by these factors. Finally, the sample size was relatively small, which might have led to overfitting in the multivariable prediction models. Therefore, we did not include more variables at the medical institutions in these models.

## CONCLUSION

Our study showed that the low proportion of OHCA patients who received bystander CPR contributed significantly to a low rate of good neurological survival in Vietnam. Upon admission, bystander CPR was an independent predictor of good neurological survival at hospital discharge. To improve the chances of good neurological survival of OHCA patients, more people should be trained to perform bystander CPR and teach others as well. A standard program for emergency first-aid training is necessary for this purpose.

## Supplementary Information




