# Supplementary material for: Impact of Bystander Cardiopulmonary Resuscitation on Out-of-Hospital Cardiac Arrest Outcome in Vietnam
Source: West J Emerg Med. 2024 Jun 14;25(4):507–20. doi: 10.5811/westjem.18413 (PMC11254151; doi:10.5811/westjem.18413)
Supplement: Supplementary file 1 [file wjem-25-507-s001.docx]

**SUPPLEMENTARY RESULTS**

**THE TITLE OF ARTICLE: THE PROVISION OF BYSTANDER CARDIOPULMONARY RESUSCITATION FOR OUT-OF-HOSPITAL CARDIAC ARREST IN A LOW- AND MIDDLE-INCOME COUNTRY: IMPACT ON OUTCOMES**

**TABLE OF CONTENTS**

**Figure S1**. Flowchart of pairwise 1:1 propensity score matching model

**Table S1**. General characteristics, management, and outcomes of non-matched cohorts of patients with out-of-hospital cardiac arrest according to the type of bystander cardiopulmonary resuscitation, Vietnam, February 2014 - December 2018

**Table S2**. General characteristics, management, and outcomes of patients with out-of-hospital cardiac arrest according to hospital admission, Vietnam, February 2014 - December 2018

**Table S3**. General characteristics, management, and outcomes of patients with out-of-hospital cardiac arrest according to hospital discharge, Vietnam, February 2014 - December 2018

**Table S4**. General characteristics, management, and outcomes of patients with out-of-hospital cardiac arrest according to neurological status, Vietnam, February 2014 - December 2018

**Table S5**. General characteristics, management, and outcomes of matched cohorts of patients with out-of-hospital cardiac arrest according to the type of bystander cardiopulmonary resuscitation, Vietnam, February 2014 - December 2018

**Table S6**. Factors associated with survival to hospital admission in patients with out-of-hospital cardiac arrest: univariable logistic regression analyses

**Table S7**. Factors associated with survival to hospital discharge in patients with out-of-hospital cardiac arrest: univariable logistic regression analyses

**Table S8**. Factors associated with good neurological survival at hospital discharge in patients with out-of-hospital cardiac arrest: univariable logistic regression analyses

**Table S9**. Factors associated with survival to hospital admission in patients with out-of-hospital cardiac arrest: multivariable logistic regression analysis (backward elimination)

**Table S10** Factors associated with survival to hospital discharge in patients with out-of-hospital cardiac arrest: multivariable logistic regression analysis (backward elimination)

**Table S11**. Factors associated with good neurological status in patients with out-of-hospital cardiac arrest: multivariable logistic regression analysis (backward elimination)

**Table S12**. Breakdown of missing data

Total patients were assessed for eligibility

n=779

Patients were eligible for analysis

n=521

Excluded patients: 258

● 31 ≤18 years

● 109 with traumatic etiology of cardiac arrest

● 30 with long pre-hospital time

● 88 with missing or unknown data

Included patients who did not receive bystander CPR, n=406

Included patients who received bystander CPR, n=115

Matched patients who received bystander CPR, n=106

Propensity score matching model: age, sex, past medical history, and etiology of OHCA^*^

Matched patients who did not receive bystander CPR, n=106

**Figure S1**. Flowchart of pairwise 1:1 propensity score matching model. ^*^**Age** (either less than 60 years or greater than or equal to 60 years), **sex** (either male or female), **past medical history** (none, heart diseases only, other diseases, such as diabetes, cancer, hypertension, renal disease, respiratory disease, hyperlipidemia, stroke, HIV, and others, or both heart diseases and other diseases), and **etiology of OHCA** (either non-cardiac, such as respiratory, drowning, electrocution, and others, or presumed cardiac). Abbreviations: **CPR**, cardiopulmonary resuscitation; **OHCA**, out-of-hospital cardiac arrest.

**Table S1**. General characteristics, management, and outcomes of non-matched cohorts of patients with out-of-hospital cardiac arrest according to the type of bystander cardiopulmonary resuscitation, Vietnam, February 2014 - December 2018

| Characteristics | All cases | No bystander CPR | Bystander CPR | p-value^a^ |
| --- | --- | --- | --- | --- |
| **Hospital participated** |  |  |  |  |
| Hospital | n=521 | n=406 | n=115 | 0.026 |
| Bach Mai, no. (%) | 396 (76.0) | 306 (75.4) | 90 (78.3) |  |
| Hue, no. (%) | 24 (4.6) | 24 (5.9) | 0 |  |
| Cho Ray, no. (%) | 101 (19.4) | 76 (18.7) | 25 (21.7) |  |
| **Patient-related** | n=521 | n=406 | n=115 |  |
| Age (year), mean (SD) | 56.71 (17.32) | 57.56 (17.17) | 53.7 (17.57) | 0.035^**^ |
| Age (year), no. (%) |  |  |  | 0.203 |
| < 60 | 290 (55.7) | 220 (54.2) | 70 (60.9) |  |
| ≥ 60 | 231 (44.3) | 186 (45.8) | 45 (39.1) |  |
| Gender, no. (%) |  |  |  | 0.522 |
| Male | 388 (74.5) | 305 (75.10 | 83 (72.2) |  |
| Female | 133 (25.5) | 101 (24.9) | 32 (27.8) |  |
| Past medical history, no. (%) | n=470 | n=364 | n=106 |  |
| Heart disease | 85 (18.1) | 60 (16.5) | 25 (23.6) | 0.095 |
| Diabetes | 64 (13.6) | 46 (12.6) | 18 (17.00 | 0.251 |
| Cancer | 38 (8.1) | 34 (9.3) | 4 (3.8) | 0.064 |
| Hypertension | 111(23.6) | 85 (23.4) | 26 (24.5) | 0.802 |
| Renal disease | 38 (8.1) | 27 (7.4) | 11 (10.4) | 0.325 |
| Respiratory disease | 75 (16.0) | 53 (14.6) | 22 (20.8) | 0.125 |
| Hyperlipidemia | 4 (0.9) | 4 (1.1) | 0 | 0.579^*^ |
| Stroke | 16 (3.4) | 15 (4.1) | 1 (0.9) | 0.137^*^ |
| HIV | 1 (0.2) | 1 (0.3) | 0 | >0.999^*^ |
| Other | 89 (18.9) | 67 (18.4) | 22 (20.8) | 0.587 |
| Past medical history | n=470 | n=364 | n=106 | 0.224 |
| None | 107 (22.8) | 83 (22.8) | 24 (22.6) |  |
| Heart disease | 24 (5.1) | 19 (5.2) | 5 (4.7) |  |
| No heart disease | 278 (59.1) | 221 (60.7) | 57 (53.8) |  |
| Both | 61 (13.0) | 41 (11.3) | 20 (18.9) |  |
| **Event-related** |  |  |  |  |
| Location type, no. (%) |  |  |  | <0.001^*^ |
| Home residence | 358 (68.7) | 304 (74.9) | 54 (47.0) |  |
| Healthcare facility | 49 (9.4) | 14 (3.4) | 35 (30.4) |  |
| In EMS/Private ambulance | 63 (12.1) | 6 (11.3) | 17 (14.8) |  |
| Industrial place | 4 (0.8) | 4 (1.0) | 0 |  |
| Nursing home | 1 (0.2) | 0 | 1 (0.9) |  |
| Place of recreation | 3 (0.6) | 2 (0.5) | 1 (0.9) |  |
| Public/Commercial building | 5 (1.0) | 4 (1.0) | 1 (0.9) |  |
| Street/Highway | 16 (3.1) | 13 (3.2) | 3 (2.6) |  |
| Transport center | 1 (0.2) | 1 (0.2) | 0 |  |
| Other | 21 (4.0) | 18 (4.4) | 3 (2.6) |  |
| Location type |  |  |  | <0.001 |
| In EMS/Private ambulance | 63 (12.1) | 46 (11.3) | 17 (14.8) |  |
| Healthcare facility | 50 (9.6) | 14 (3.4) | 36 (31.3) |  |
| Home residence | 358 (68.7) | 304 (74.9) | 54 (47.0) |  |
| Public area | 50 (9.6 | 42 (10.3 | 8 (7.0 |  |
| Time of the day, no. (%) n=320 | 181/320 (56.6) | 125/236 (53.0) | 56/84 (66.7) | 0.030 |
| Arrest witnessed by, no. (%) | n=520 | n=405 | n=115 | <0.001 |
| Not witnessed | 110 (21.2) | 108 (26.7) | 2 (1.7) |  |
| Bystander (Lay person) | 22 (4.2) | 16 (4.0) | 6 (5.2) |  |
| Bystander (Family) | 72 (13.8) | 19 (4.7) | 53 (46.1) |  |
| Bystander (Healthcare provider) | 259 (49.8) | 229 (56.5) | 30 (26.1) |  |
| EMS/Private ambulance | 57 (11.0) | 33 (8.1) | 24 (20.9) |  |
| Witnessed arrest | n=520 | n=405 | n=115 | <0.001 |
| No | 110 (21.2) | 108 (26.7) | 2 (1.7) |  |
| Yes | 410 (78.8) | 297 (73.30 | 113 (98.3) |  |
| Arrest witnessed by | n=520 | n=405 | n=115 | <0.001 |
| None | 110 (21.2) | 108 (26.7) | 2 (1.7) |  |
| Bystander | 281 (54.0) | 245 (60.5) | 36 (31.3) |  |
| Healthcare provider | 72 (13.8) | 19 (4.7) | 53 (46.1) |  |
| EMS/Private ambulance | 57 (11.0) | 33 (8.1) | 24 (20.9) |  |
| Etiology of OHCA, no. (%) |  |  |  | 0.257^*^ |
| Presumed cardiac etiology | 234 (44.9) | 184 (45.3) | 50 (43.5) |  |
| Respiratory | 124 (23.8) | 94 (23.2) | 30 (26.1) |  |
| Drowning | 6 (1.2) | 3 (0.7) | 3 (2.6) |  |
| Electrocution | 8 (1.5) | 8 (2.0) | 0 |  |
| Other | 149 (28.6) | 117 (28.8) | 32 (27.8) |  |
| Etiology of OHCA |  |  |  | 0.726 |
| Non-cardiac | 287 (55.1) | 222 (54.7) | 65 (56.5) |  |
| Presumed cardiac | 234 (44.9) | 184 (45.3) | 50 (43.5) |  |
| First arrest rhythm, no. (%) |  |  |  | 0.089^*^ |
| VT | 11 (8.1) | 4 (5.3) | 7 (11.9) |  |
| VF | 29 (21.5 | 22 (28.9 | 7 (11.9) |  |
| Unknown shockable rhythm | 53 (39.3 | 25 (32.9 | 28 (47.5) |  |
| Unknown unshockable rhythm | 20 (14.8 | 13 (17.1) | 7 (11.9) |  |
| PEA | 5 (3.7) | 2 (2.6) | 3 (5.1) |  |
| Asystole | 17 (12.6) | 10 (13.2) | 7 (11.9) |  |
| First arrest rhythm | n=135 | n=76 | n=59 | 0.611 |
| Unshockable rhythm | 42 (31.1) | 25 (32.9) | 17 (28.8) |  |
| Shockable rhythm | 93 (68.9) | 51 (67.1) | 42 (71.2) |  |
| **System-related** |  |  |  |  |
| Types of pre-hospital transportation, no. (%) |  |  |  | <0.001 |
| EMS | 42 (8.1) | 26 (6.4) | 16 (13.9) |  |
| Private ambulance | 223 (42.8) | 142 (35.0) | 81 (70.4) |  |
| Private or public transport | 256 (49.1) | 238 (58.6) | 18 (15.7) |  |
| Resuscitation attempted by EMS/private ambulance, no. (%) | 133/262 (50.8) | 75/168 (44.6) | 58/94 (61.7) | 0.008 |
| Time to initiation of CPR (min), mean (SD) | 7.33/87 (8.70) | 9.14/48 (5.59) | 5.10/39 (11.11) | <0.001^***^ |
| Time to defibrillation at scene (min), mean (SD) | 9.00/36 (6.21) | 9.72/23 (5.05) | 7.74/13 (7.94) | 0.125^***^ |
| **Therapy-related** |  |  |  |  |
| Pharmacotherapy, no. (%) |  |  |  |  |
| Epinephrine (at scene) | 122 (23.4) | 67 (16.5) | 55 (47.8) | <0.001 |
| Epinephrine (at ED) | 480 (92.1) | 374 (92.1) | 106 (92.2) | >0.999 |
| Pre-hospital intervention, no. (%) |  |  |  |  |
| Pre-hospital defibrillation | 43/135 (31.9) | 29/76 (38.2) | 14/59 (23.7) | 0.074 |
| Bystander AED applied | 14/262 (5.3) | 7/168 (4.2) | 7/94 (7.4) | 0.257 |
| ED defibrillation performed, no. (%) | 68 (13.1) | 48 (11.8) | 20 (17.4) | 0.118 |
| Pre-hospital advanced airway, no. (%) | 108 (20.7) | 62 (15.3) | 46 (40.0) | <0.001 |
| Pre-hospital advanced airway techniques, no. (%) | n=108 | n=62 | n=46 | >0.999^*^ |
| Oral/Nasal ET | 106 (98.1) | 60 (96.8) | 46 (100.0) |  |
| LMA | 1 (0.9) | 1 (1.6) | 0 |  |
| Other | 1 (0.9) | 1 (1.6) | 0 |  |
| Advanced airway used at ED, no. (%) | 297 (57.0) | 241 (59.4) | 56 (48.7) | 0.041 |
| Advanced airway techniques used at ED, no. (%) | n=297 | n=241 | n=56 | 0.467^*^ |
| Oral/Nasal ET | 294 (99.0) | 239 (99.2) | 55 (98.2) |  |
| LMA | 1 (0.3) | 1 (0.4) | 0 |  |
| Other | 2 (0.7) | 1 (0.4) | 1 (1.8) |  |
| Immediate coronary angiography on admission to hospital, no. (%) |  |  |  |  |
| Emergency PCI performed | 23 (4.4) | 18 (4.4) | 5 (4.3) | 0.969 |
| Emergency CABG performed | 2 (0.4) | 2 (0.5) | 0 | >0.999^*^ |
| Post-resuscitation care, no. (%) |  |  |  |  |
| ECMO therapy initiated | 7/519 (1.30) | 5/404 (1.2) | 2/115 (1.7) | 0.654^*^ |
| Hypothermia therapy initiated | 78 (15.0) | 53 (13.1) | 25 (21.7) | 0.021 |
| **Outcomes** |  |  |  |  |
| ROSC, no. (%) |  |  |  |  |
| ROSC at scene/en-route | 98 (18.8) | 57 (14.0) | 41 (35.7) | <0.001 |
| ROSC at ED | 113 (21.7) | 81 (20.00 | 32 (27.8) | 0.070 |
| Outcome of patient at ED, no. (%) |  |  |  | 0.064 |
| Died in ED | 425 (81.6) | 338 (83.3) | 87 (75.7) |  |
| Admitted | 96 (18.4) | 68 (16.7) | 28 (24.3) |  |
| Patient status, no. (%) |  |  |  | 0.137^*^ |
| Died in the hospital | 41 (7.9) | 31 (7.6) | 10 (8.7) |  |
| Remains in hospital at 30th day post arrest | 6 (1.2) | 5 (1.2) | 1 (0.9) |  |
| Discharged alive | 49 (9.4) | 32 (7.90) | 17 (14.8) |  |
| Post arrest CPC 1 or 2, n (%) | 33 (6.3) | 19 (4.7) | 14 (12.2) | 0.004 |
| ^a^ The comparison between patients who did not receive bystander CPR and who received bystander CPR, using the Chi-squared test; ^*^Fisher’s exact test; ^**^Independent-samples T-test; ^***^Mann-Whitney U test. Abbreviations: **AED**, automatic external defibrillation; **CABG**, coronary artery bypass grafting; **CPC**, cerebral performance category; **CPR**, cardiopulmonary resuscitation; **ECMO**, extracorporeal membrane oxygenation; **ED**, emergency department; **EMS**, emergency medical services; **ET**, endotracheal; **LMA**, laryngeal mask airway; **OHCA**, out-of-hospital cardiac arrest; **PCI**, percutaneous coronary intervention; **PEA**, pulseless electrical activity; **ROSC**, return of spontaneous circulation; **SD**, standard deviation; **Time of the day**, period from 8:00 to 20:00 hour; **VF**, ventricular fibrillation; **VT**, ventricular tachycardia. | | | | |

**Table S2**. General characteristics, management, and outcomes of patients with out-of-hospital cardiac arrest according to hospital admission, Vietnam, February 2014 - December 2018

| Characteristics | All cases  (n=521) | Died in ED (n=425) | Admitted  (n=96) | p-value^a^ |
| --- | --- | --- | --- | --- |
| **Hospital participated** |  |  |  |  |
| Hospital | n=521 | n=425 | n=96 | 0.134 |
| Bach Mai, no. (%) | 396 (76.0) | 324 (76.2) | 72 75.0) |  |
| Hue, no. (%) | 24 (4.6) | 16 (3.8) | 8 (8.3) |  |
| Cho Ray, no. (%) | 101 (19.4) | 85 (20.0) | 16 (16.7) |  |
| **Patient-related** | n=521 | n=425 | n=96 |  |
| Age (year), mean (SD) | 56.71 (17.32) | 58.46 16.66) | 48.96 (18.12) | <0.001^**^ |
| Age (year) |  |  |  | <0.001 |
| < 60, no. (%) | 290 (55.7) | 220 (51.8) | 70 (72.9) |  |
| ≥ 60, no. (%) | 231 (44.3) | 205 (48.2) | 26 (27.1) |  |
| Gender, no. (%) |  |  |  | 0.052 |
| Male | 388 (74.5) | 309 (72.7) | 79 (82.3) |  |
| Female | 133 (25.5) | 116 (27.3 | 17 (17.7) |  |
| Past medical history, no. (%) | n=470 | n=382 | n=88 |  |
| Heart disease | 85 (18.1) | 72 (18.8) | 13 (14.8) | 0.371 |
| Diabetes | 64 (13.6) | 51 (13.4) | 13 (14.8) | 0.726 |
| Cancer | 38 (8.1) | 36 (9.4) | 2 (2.3) | 0.027 |
| Hypertension | 111(23.6) | 91 (23.8) | 20 (22.7) | 0.827 |
| Renal disease | 38 (8.1) | 37 (9.7) | 1 (1.1) | 0.008 |
| Respiratory disease | 75 (16.0) | 50 (13.1) | 25 928.4) | <0.001 |
| Hyperlipidemia | 4 (0.9) | 2 (0.5) | 2 (2.3) | 0.161^*^ |
| Stroke | 16 (3.4) | 15 (3.9) | 1 (1.1) | 0.327^*^ |
| HIV | 1 (0.2) | 1 (0.3) | 0 | >0.999^*^ |
| Other | 89 (18.9) | 87 (22.8) | 2 (2.3) | <0.001 |
| Past medical history | n=470 | n=382 | n=88 | <0.001 |
| None | 107 (22.8) | 71 (18.6) | 36 (40.9) |  |
| Heart disease | 24 (5.1) | 23 (6.0) | 1 (1.1) |  |
| No heart disease | 278 (59.1) | 239 (62.6) | 39 (44.3) |  |
| Both | 61 (13.0) | 49 (12.8) | 12 (13.6) |  |
| **Event-related** |  |  |  |  |
| Location type, no. (%) | n=521 | n=425 | n=96 | 0.010^*^ |
| Home residence | 358 (68.7) | 285 (67.1) | 73 (76.0) |  |
| Healthcare facility | 49 (9.4) | 44 (10.4) | 5 (5.2) |  |
| In EMS/Private ambulance | 63 (12.1) | 58 (13.6) | 5 (5.2) |  |
| Industrial place | 4 (0.8) | 2 (0.5) | 2 (2.1) |  |
| Nursing home | 1 (0.2) | 0 | 1 (1.0) |  |
| Place of recreation | 3 (0.6) | 1 (0.2) | 2 (2.1) |  |
| Public/Commercial building | 5 (1.0) | 4 (0.9) | 1 (1.0) |  |
| Street/Highway | 16 (3.1) | 12 (2.8) | 4 (4.2) |  |
| Transport center | 1 (0.2) | 1 (0.2) | 0 |  |
| Other | 21 (4.0) | 18 (4.2) | 3 (3.1) |  |
| Location type | n=521 | n=425 | n=96 | 0.047 |
| In EMS/Private ambulance | 63 (12.1) | 58 (13.6) | 5 (5.2) |  |
| Healthcare facility | 50 (9.6) | 44 (10.4) | 6 (6.2) |  |
| Home residence | 358 (68.7) | 285 (67.1) | 73 (76.0) |  |
| Public area | 50 (9.6) | 38 (8.9) | 12 (12.5) |  |
| Time of the day, no. (%) | 181/320 (56.6) | 127/238 (53.4) | 54/82 (65.9) | 0.049 |
| Arrest witnessed by, no. (%) | n=520 | n=424 | n=96 | 0.015 |
| Not witnessed | 110 (21.2) | 102 (24.1) | 8 (8.3) |  |
| Bystander (Lay person) | 22 (4.2) | 17 (4.0) | 5 (5.2) |  |
| Bystander (Family) | 72 (13.8) | 59 (13.9) | 13 (13.5) |  |
| Bystander (Healthcare provider) | 259 (49.8) | 201 (47.4) | 58 (60.4) |  |
| EMS/Private ambulance | 57 (11.0) | 45 (10.6) | 12 (12.5) |  |
| Witnessed arrest | n=520 | n=424 | n=96 | 0.001 |
| No | 110 (21.2) | 101 924.1) | 8 (8.3) |  |
| Yes | 410 (78.8) | 322 (75.9) | 88 (991.7) |  |
| Arrest witnessed by | n=520 | n=424 | n=96 | 0.006 |
| None | 110 (21.2) | 102 (24.1) | 8 (8.3) |  |
| Bystander | 281 (54.0) | 218 (51.4) | 63 (65.6) |  |
| Healthcare provider | 72 (13.8) | 59 (13.9) | 13 (13.5) |  |
| EMS/Private ambulance | 57 (11.0) | 45 (10.6) | 12 (12.5) |  |
| Etiology of OHCA, no. (%) | n=521 | n=425 | n=96 | <0.001^*^ |
| Presumed cardiac etiology | 234 (44.9) | 187 (44.0) | 47 (49.0) |  |
| Respiratory | 124 (23.8) | 89 (20.9) | 35 (36.5) |  |
| Drowning | 6 (1.2) | 5 (1.2) | 1 (0.1) |  |
| Electrocution | 8 (1.5) | 3 (0.7) | 5 (5.2) |  |
| Other | 149 (28.6) | 141 (33.2) | 8 (8.3) |  |
| Etiology of OHCA | n=521 | n=425 | n=96 | 0.378 |
| Non-cardiac | 287 (55.1) | 238 (56.0) | 49 (51.0) |  |
| Presumed cardiac | 234 (44.9) | 187 (44.0) | 47 (49.0) |  |
| First arrest rhythm, no. (%) | n=135 | n=71 | n=64 | <0.001 |
| VT | 11 (8.1) | 5 (7.0) | 6 (9.4) |  |
| VF | 29 (21.5) | 6 (8.5) | 23 (35.9) |  |
| Unknown shockable rhythm | 53 (39.3) | 44 (62.0) | 9 (14.1) |  |
| Unknown unshockable rhythm | 20 (14.8) | 2 (2.8) | 18 (28.1) |  |
| PEA | 5 (3.7) | 5 (7.0) | 0 |  |
| Asystole | 17 (12.6) | 9 (12.7) | 8 (12.5) |  |
| First arrest rhythm | n=135 | n=71 | n=64 | 0.023 |
| Unshockable rhythm | 42 (31.1) | 16 (22.5) | 26 (40.6) |  |
| Shockable rhythm | 93 (68.9) | 55 (77.5) | 38 (59.4) |  |
| **System-related** |  |  |  |  |
| Pre-hospital transport, no. (%) | n=521 | n=425 | n=96 | <0.001 |
| EMS | 42 (8.1) | 31 (7.3) | 11 (11.5) |  |
| Private ambulance | 223 (42.8) | 161 (37.9) | 62 (64.6) |  |
| Private or public transport | 256 (49.1) | 233 (54.8) | 23 (24.0) |  |
| Resuscitation attempted by EMS/private ambulance, no. (%) | 133/262 (50.8) | 70/189 (37.0) | 63/73 (86.3) | <0.001 |
| Time to initiation of CPR (min), mean (SD) | 7.33/87 (8.70) | 5.76/28 (12.77) | 8.07/59 95.87) | 0.002^***^ |
| Time to defibrillation at scene (min), mean (SD) | 9.00/36 (6.21) | 4.87/7 (3.88) | 10.0/29 (6.30) | 0.037^***^ |
| **Therapy-related** |  |  |  |  |
| Pharmacotherapy, no. (%) | n=521 | n=425 | n=96 |  |
| Epinephrine (at scene) | 122 (23.4) | 61 (14.4) | 61 (63.5) | <0.001 |
| Epinephrine (at ED) | 480 (92.1) | 31 (7.3) | 10 (10.4) | 0.305 |
| Pre-hospital intervention, no. (%) | n=521 | n=425 | n=96 |  |
| Bystander CPR | 115 (22.1) | 87 (20.5) | 28 (29.2) | 0.064 |
| Pre-hospital defibrillation | 43/135 (31.9) | 12/71 (16.9) | 31/64 (48.4) | <0.001 |
| Bystander AED applied | 14/262 (5.3) | 8/189 (4.2) | 6/73 (8.2) | 0.224^*^ |
| ED defibrillation performed, no. (%) | 68 (13.1) | 44 (10.4) | 24 (25.0) | <0.001 |
| Pre-hospital advanced airway, no. (%) | 108 (20.7) | 46 (10.8) | 62 (64.6) | <0.001 |
| Pre-hospital advanced airway techniques, no. (%) | n=108 | n=46 | n=62 | 0.179^*^ |
| Oral/Nasal ET | 106 (98.1) | 44 (95.7) | 62 (100.0) |  |
| LMA | 1 (0.9) | 1 (2.2) | 0 |  |
| Other | 1 (0.9) | 1 (2.2) | 0 |  |
| Advanced airway used at ED, no. (%) | 297 (57.0) | 264 (62.1) | 33 (34.4) | <0.001 |
| Advanced airway techniques used at ED, no. (%) | n=297 | n=264 | n=33 | >0.999^*^ |
| Oral/Nasal ET | 294 (99.0) | 261 (98.9) | 33 (100.0) |  |
| LMA | 1 (0.3) | 1 (0.4) | 0 |  |
| Other | 2 (0.7) | 2 (0.8) | 0 |  |
| Immediate coronary angiography on admission to hospital, no. (%) | n=521 | n=425 | n=96 |  |
| Emergency PCI performed | 23 (4.4) | 5 (1.2) | 18 (18.8) | <0.001^*^ |
| Emergency CABG performed | 2 (0.4) | 1 (0.2) | 1 (1.0) | 0.335^*^ |
| Post-resuscitation care, no. (%) | n=521 | n=425 | n=96 |  |
| ECMO therapy initiated | 7/519 (1.30) | 5/423 (1.2) | 2/96 (2.1) | 0.619^*^ |
| Hypothermia therapy initiated | 78 (15.0) | 12 (2.8) | 66 (68.8) | <0.001 |
| **Outcomes** |  |  |  |  |
| ROSC, no. (%) | n=521 | n=425 | n=96 |  |
| ROSC at scene/en-route | 98 (18.8) | 35 (8.2) | 63 (65.6) | <0.001 |
| ROSC at ED | 113 (21.7) | 69 (16.2) | 44 (45.8) | <0.001 |
| Patient status, no. (%) | n=521 | n=425 | n=96 | <0.001 |
| Died in the hospital | 41 (7.9) | 0 | 41 (42.7) |  |
| Remains in hospital at 30th day post arrest | 6 (1.2) | 0 | 6 (6.3) |  |
| Discharged alive | 49 (9.4) | 0 | 49 (51.0) |  |
| Post arrest CPC 1 or 2, n (%) | 33 (6.3) | 0 | 33 (34.4) | <0.001 |
| ^a^ The comparison between patients who died in the ED and who survived to hospital admission, using the Chi-squared test; ^*^Fisher’s exact test; ^**^Independent-samples T-test; ^***^Mann-Whitney U test. Abbreviations: **AED**, automatic external defibrillation; **CABG**, coronary artery bypass grafting; **CPC**, cerebral performance category; **CPR**, cardiopulmonary resuscitation; **ECMO**, extracorporeal membrane oxygenation; **ED**, emergency department; **EMS**, emergency medical services; **ET**, endotracheal; **LMA**, laryngeal mask airway; **OHCA**, out-of-hospital cardiac arrest; **PCI**, percutaneous coronary intervention; **PEA**, pulseless electrical activity; **ROSC**, return of spontaneous circulation; **SD**, standard deviation; **Time of the day**, period from 8:00 to 20:00 hour; **VF**, ventricular fibrillation; **VT**, ventricular tachycardia. | | | | |

**Table S3**. General characteristics, management, and outcomes of patients with out-of-hospital cardiac arrest according to hospital discharge, Vietnam, February 2014 - December 2018

| Characteristics | All cases  (n=521) | Not hospital discharge (n=472) | Hospital discharge  (n=49) | p-value^a^ |
| --- | --- | --- | --- | --- |
| **Hospital participated** |  |  |  |  |
| Hospital |  |  |  | 0.003 |
| Bach Mai hospital, no. (%) | 396 (76.0) | 349 (73.9) | 47 (95.9) |  |
| Hue hospital, no. (%) | 24 (4.6) | 24 (5.1) | 0 |  |
| Cho Ray hospital, no. (%) | 101 (19.4) | 99 (21.0) | 2 (2.1) |  |
| **Patient-related** |  |  |  |  |
| Age, mean (SD) | 56.71 (17.32) | 58.11 (16.77) | 43.2 (16.78) | <0.001^**^ |
| Age (year) |  |  |  | <0.001 |
| < 60, no. (%) | 29 (55.7) | 251 (53.2) | 39 (79.6) |  |
| ≥ 60, no. (%) | 231 (44.3) | 221 (46.8) | 10 (20.4) |  |
| Gender, no. (%) |  |  |  | 0.058 |
| Male | 388 (74.5) | 346 (73.3) | 42 (85.7) |  |
| Female | 133 (25.5) | 126 (26.7) | 7 1(4.3) |  |
| Past medical history, no. (%) | n=470 |  |  |  |
| Heart disease | 85 (18.1) | 83 (19.7) | 2 (4.2) | 0.008 |
| Diabetes | 64 (13.6) | 59 (14.0) | 5 (10.4) | 0.495 |
| Cancer | 38 (8.1) | 37 (8.8) | 1 (2.1) | 0.158^*^ |
| Hypertension | 111(23.6) | 102 (24.2) | 9 (18.8) | 0.402 |
| Renal disease | 38 (8.1) | 37 (8.8) | 1 (2.1) | 0.158^*^ |
| Respiratory disease | 75 (16.0) | 61 (14.5) | 14 (29.2) | 0.008 |
| Hyperlipidemia | 4 (0.9) | 4 (0.9) | 0 | >0.999^*^ |
| Stroke | 16 (3.4) | 16 (3.8) | 0 | 0.391^*^ |
| HIV | 1 (0.2) | 1 (0.2) | 0 | >0.999^*^ |
| Other | 89 (18.9) | 89 (21.1) | 0 | <0.001 |
| Past medical history | n=470 | n=422 | n=48 | <0.001 |
| None | 107 (22.8) | 81 19.2) | 26 54.2) |  |
| Heart disease | 24 (5.1) | 24 5.7) | 0 |  |
| No heart disease | 278 (59.1) | 258 61.1) | 20 41.7) |  |
| Both | 61 (13.0) | 59 14.0) | 2 4.2) |  |
| **Event-related** |  |  |  |  |
| Location type, no. (%) |  |  |  | 0.031^*^ |
| Home residence | 358 (68.7) | 321 (68.0) | 37 (75.5) |  |
| Healthcare facility | 49 (9.4) | 47 (10.0) | 2 (4.1) |  |
| In EMS/Private ambulance | 63 (12.1) | 60 (12.7) | 3 (6.1) |  |
| Industrial place | 4 (0.8) | 2 (0.4) | 2 (4.1) |  |
| Nursing home | 1 (0.2) | 0 | 1 (2.0) |  |
| Place of recreation | 3 (0.6) | 2 (0.4) | 1 (2.0) |  |
| Public/Commercial building | 5 (1.0) | 5 (1.1) | 0 |  |
| Street/Highway | 16 (3.1) | 15 (3.2) | 1 (2.0) |  |
| Transport center | 1 (0.2) | 1 (0.2) | 0 |  |
| Other | 21 (4.0) | 19 (4.0) | 2 (4.1) |  |
| Location type |  |  |  | 0.422^*^ |
| In EMS/Private ambulance | 63 (12.1) | 60 12.7 | 3 6.1 |  |
| Healthcare facility | 50 (9.6) | 47 10.0 | 3 6.1 |  |
| Home residence | 358 (68.7) | 321 68.0 | 37 75.5 |  |
| Public area | 50 (9.69) | 44 9.3 | 6 12.2 |  |
| Time of the day, no. (%) | 181/320 (56.6) | 147 54.0 | 34 70.8 |  |
| Arrest witnessed by, no. (%) | n=520 | n=471 | n=49 | 0.098 |
| Not witnessed | 110 (21.2) | 107 (22.7) | 3 (6.1) |  |
| Bystander (Lay person) | 22 (4.2) | 20 (4.2) | 2 (4.1) |  |
| Bystander (Family) | 72 (13.8) | 65 (13.8) | 7 (14.3) |  |
| Bystander (Healthcare provider) | 259 (49.8) | 228 (48.4) | 31 (63.3) |  |
| EMS/Private ambulance | 57 (11.0) | 51 (10.8) | 6 (12.2) |  |
| Witnessed arrest | n=520 | n=471 | n=49 | 0.007 |
| No | 110 (21.2) | 107 (22.7) | 3 (6.1) |  |
| Yes | 410 (78.8) | 364 (77.3) | 46 (93.9) |  |
| Arrest witnessed by | n=520 | n=471 | n=49 | 0.054 |
| None | 110 (21.2) | 107 (22.7) | 3 (6.1) |  |
| Bystander | 281 (54.0) | 248 (52.7) | 33 (67.3) |  |
| Healthcare provider | 72 (13.8) | 65 (13.8) | 7 (14.3) |  |
| EMS/Private ambulance | 57 (11.0) | 51 (10.8) | 6 (12.2) |  |
| Etiology of OHCA, no. (%) |  |  |  | <0.001^*^ |
| Presumed cardiac etiology | 234 (44.9) | 208 (44.1) | 26 (53.1) |  |
| Respiratory | 124 (23.8) | 104 (22.0) | 20 (40.8) |  |
| Drowning | 6 (1.2) | 6 (1.3) | 0 |  |
| Electrocution | 8 (1.5) | 6 (1.3) | 2 (4.1) |  |
| Other | 149 (28.6) | 148 (31.4) | 1 (2.0) |  |
| Etiology of OHCA |  |  |  | 0.228 |
| Non-cardiac | 287 (55.1) | 264 (55.9) | 23 (46.9) |  |
| Presumed cardiac | 234 (44.9) | 208 (44.1) | 26 (53.1) |  |
| First arrest rhythm, no. (%) | n=135 | n=97 | n=38 | <0.001^*^ |
| VT | 11 (8.1) | 10 (10.3) | 1 (2.6) |  |
| VF | 29 (21.5) | 10 (10.3) | 19 (50.0) |  |
| Unknown shockable rhythm | 53 (39.3) | 50 (51.5) | 3 (7.9) |  |
| Unknown unshockable rhythm | 20 (14.8) | 9 (9.3) | 11 (28.9) |  |
| PEA | 5 (3.7) | 5 (5.2) | 0 |  |
| Asystole | 17 (12.6) | 13 (13.4) | 4 (10.5) |  |
| First arrest rhythm | n=135 | n=97 | n=38 | 0.189 |
| Unshockable rhythm | 42 (31.1) | 27 (27.8) | 15 (39.5) |  |
| Shockable rhythm | 93 (68.9) | 70 (72.2) | 23 (60.5) |  |
| **System-related** |  |  |  |  |
| Types of pre-hospital transportation, no. (%) |  |  |  | <0.001 |
| EMS | 42 (8.1) | 41 (8.7) | 1 (2.0) |  |
| Private ambulance | 223 (42.8) | 185 (39.2) | 38 (77.6) |  |
| Private or public transport | 256 (49.1) | 246 (52.1) | 10 (20.4) |  |
| Resuscitation attempted by EMS/private ambulance, no. (%) | 133/262 (50.8) | 95/223 (42.6) | 38/39 (97.4) | <0.001 |
| Time to initiation of CPR (min), mean (SD) | 7.33/87 (8.70) | 7.25/50 (10.57) | 7.44/37 (5.35) | 0.331^***^ |
| Time to defibrillation at scene (min), mean (SD) | 9.00/36 (6.21) | 5.49/16 (4.30) | 11.81/20 (6.14) | 0.002^***^ |
| **Therapy-related** |  |  |  |  |
| Pharmacotherapy, no. (%) |  |  |  |  |
| Epinephrine (at scene) | 122 (23.4) | 85 (18.0) | 37 (75.5) | <0.001 |
| Epinephrine (at ED) | 480 (92.1) | 432 (91.5) | 48 (98.0) | 0.160^*^ |
| Pre-hospital intervention, no. (%) |  |  |  |  |
| Bystander CPR | 115 22.1 | 98 (20.8) | 17 (34.7) | 0.025 |
| Pre-hospital defibrillation | 43/135 (31.9) | 22/97 (22.7) | 21/38 (55.3) | <0.001 |
| Bystander AED applied | 14/262 (5.3) | 9/223 (4.0) | 5/39 (12.8) | 0.041^*^ |
| ED defibrillation performed, no. (%) | 68 (13.1) | 59 (12.5) | 9 (18.4) | 0.246 |
| Pre-hospital advanced airway, no. (%) | 108 (20.7) | 71 (15.0) | 37 (75.5) | <0.001 |
| Pre-hospital advanced airway techniques, no. (%) | n=108 | n=71 | n=37 | >0.999^*^ |
| Oral/Nasal ET | 106 (98.1) | 69 (97.2) | 37 (100.0) |  |
| LMA | 1 (0.9) | 1 (1.4) | 0 |  |
| Other | 1 (0.9) | 1 (1.4) | 0 |  |
| Advanced airway used at ED, no. (%) | 297 (57.0) | 285 (60.4) | 12 (24.5) | <0.001 |
| Advanced airway techniques used at ED, no. (%) | n=297 | n=285 | n=12 | >0.999^*^ |
| Oral/Nasal ET | 294 (99.0) | 282 (98.9) | 12 (100.0) |  |
| LMA | 1 (0.3) | 1 (0.4) | 0 |  |
| Other | 2 (0.7) | 2 (0.7) | 0 |  |
| Immediate coronary angiography on admission to hospital, no. (%) |  |  |  |  |
| Emergency PCI performed | 23 (4.4) | 15 3.2) | 8 16.3) | 0.001^*^ |
| Emergency CABG performed | 2 (0.4) | 2 0.4) | 0 | >0.999^*^ |
| Post-resuscitation care, no. (%) |  |  |  |  |
| ECMO therapy initiated | 7/519 (1.3) | 5 (1.1) | 2 (4.1) | 0.135^*^ |
| Hypothermia therapy initiated | 78 (15.0) | 32 (6.8) | 46 (93.9) | <0.001 |
| **Outcomes** |  |  |  |  |
| ROSC, no. (%) |  |  |  |  |
| ROSC at scene/en-route | 98 (18.8) | 60 (12.7) | 38 (77.6) | <0.001 |
| ROSC at ED | 113 (21.7) | 95 (20.1) | 18 (36.7) | 0.007 |
| Outcome of patient at ED, no. (%) |  |  |  | <0.001 |
| Died in ED | 425 (81.6) | 425 (90.0) | 0 |  |
| Admitted | 96 (18.4) | 47 (10.0) | 49 (100.0) |  |
| Post arrest CPC 1 or 2, n (%) | 33 (6.3) | 0 | 33 (67.3) | <0.001^*^ |
| ^a^ The comparison between patients who died in the hospital and who survived to hospital discharge, using the Chi-squared test; ^*^Fisher’s exact test; ^**^Independent-samples T-test; ^***^Mann-Whitney U test. Abbreviations: **AED**, automatic external defibrillation; **CABG**, coronary artery bypass grafting; **CPC**, cerebral performance category; **CPR**, cardiopulmonary resuscitation; **ECMO**, extracorporeal membrane oxygenation; **ED**, emergency department; **EMS**, emergency medical services; **ET**, endotracheal; **LMA**, laryngeal mask airway; **OHCA**, out-of-hospital cardiac arrest; **PCI**, percutaneous coronary intervention; **PEA**, pulseless electrical activity; **ROSC**, return of spontaneous circulation; **SD**, standard deviation; **Time of the day**, period from 8:00 to 20:00 hour; **VF**, ventricular fibrillation; **VT**, ventricular tachycardia. | | | | |

**Table S4**. General characteristics, management, and outcomes of patients with out-of-hospital cardiac arrest according to neurological status, Vietnam, February 2014 - December 2018

| Characteristics | All cases  (n=521) | Post arrest CPC 3 to 5 (n=488) | Post arrest CPC 1 or 2 (n=33) | p-value^a^ |
| --- | --- | --- | --- | --- |
| **Hospital participated** |  |  |  |  |
| Hospital |  |  |  | 0.042 |
| Bach Mai hospital, no. (%) | 396 (76.0) | 365 (74.8) | 31 (93.9) |  |
| Hue hospital, no. (%) | 24 (4.6) | 24 (4.9) | 0 |  |
| Cho Ray hospital, no. (%) | 101 (19.4) | 99 (20.3) | 2 (6.1) |  |
| **Patient-related** |  |  |  |  |
| Age, mean (SD) | 56.71 (17.32) | 57.69 (16.95) | 42.21 (16.42) | <0.001^**^ |
| Age (year) |  |  |  | 0.002 |
| < 60, no. (%) | 290 (55.7) | 263 (53.9) | 27 (81.8) |  |
| ≥ 60, no. (%) | 231 (44.3) | 225 (46.1) | 6 (18.2) |  |
| Gender, no. (%) |  |  |  | 0.158 |
| Male | 388 (74.5) | 360 (73.8) | 28 (84.8) |  |
| Female | 133 (25.5) | 128 (26.2) | 5 (15.2) |  |
| Past medical history, no. (%) | n=470 | n=437 | n=33 |  |
| Heart disease | 85 (18.1) | 84 (19.2) | 1 (3.0) | 0.020 |
| Diabetes | 64 (13.6) | 61 (14.0) | 3 (9.1) | 0.601^*^ |
| Cancer | 38 (8.1) | 37 (8.5) | 1 (3.0) | 0.503^*^ |
| Hypertension | 111(23.6) | 106 (24.3) | 5 (15.2) | 0.235 |
| Renal disease | 38 (8.1) | 38 (8.7) | 0 | 0.096^*^ |
| Respiratory disease | 75 (16.0) | 64 (14.6) | 11 (33.3) | 0.005 |
| Hyperlipidemia | 4 (0.9) | 4 (0.9) | 0 | >0.999^*^ |
| Stroke | 16 (3.4) | 16 (3.7) | 0 | 0.617^*^ |
| HIV | 1 (0.2) | 1 (0.2) | 0 | >0.999^*^ |
| Other | 89 (18.9) | 89 (20.4) | 0 | 0.004 |
| Past medical history | n=470 | n=437 | n=33 | <0.001^*^ |
| None | 107 (22.8) | 88 (20.1) | 19 (57.6) |  |
| Heart disease | 24 (5.1) | 24 (5.5) | 0 |  |
| No heart disease | 278 (59.1) | 265 (60.6) | 13 (39.4) |  |
| Both | 61 (13.0) | 60 (13.7) | 1 (3.0) |  |
| **Event-related** |  |  |  |  |
| Location type, no. (%) |  |  |  | 0.097^*^ |
| Home residence | 358 (68.7) | 334 (68.4) | 24 (72.7) |  |
| Healthcare facility | 49 (9.4) | 47 (9.6) | 2 (6.1) |  |
| In EMS/Private ambulance | 63 (12.1) | 60 (12.3) | 3 (9.1) |  |
| Industrial place | 4 (0.8) | 3 (0.6) | 1 (3.0) |  |
| Nursing home | 1 (0.2) | 0 | 1 (3.0) |  |
| Place of recreation | 3 (0.6) | 2 (0.4) | 1 (3.0) |  |
| Public/Commercial building | 5 (1.0) | 5 (1.0) | 0 |  |
| Street/Highway | 16 (3.1) | 16 (3.3) | 0 |  |
| Transport center | 1 (0.2) | 1 (0.2) | 0 |  |
| Other | 21 (4.0) | 20 (4.1) | 1 (3.0) |  |
| Location type |  |  |  |  |
| In EMS/Private ambulance | 63 (12.1) | 60 (12.3) | 3 (9.1) | 0.987^*^ |
| Healthcare facility | 50 (9.6) | 47 (9.6) | 3 (9.1) |  |
| Home residence | 358 (68.7) | 334 (68.4) | 24 (72.7) |  |
| Public area | 50 (9.6) | 47 (9.6) | 3 (9.1) |  |
| Time of the day, no. (%) | 181/320 (56.6) | 156/287 (54.4) | 25/33 (75.8) | 0.019 |
| Arrest witnessed by, no. (%) | n=520 | n=487 | n=33 | 0.117^*^ |
| Not witnessed | 110 (21.2) | 108 (22.2) | 2 (6.1) |  |
| Bystander (Lay person) | 22 (4.2) | 21 (4.3) | 1 (3.0) |  |
| Bystander (Family) | 72 (13.8) | 66 (13.6) | 6 (18.2) |  |
| Bystander (Healthcare provider) | 259 (49.8) | 241 (49.5) | 18 (54.5) |  |
| EMS/Private ambulance | 57 (11.0) | 51 (10.5) | 6 (18.2) |  |
| Witnessed arrest | n=520 | n=487 | n=33 | 0.028 |
| No | 110 (21.2) | 108 (22.2) | 2 (6.1) |  |
| Yes | 410 (78.8) | 379 (77.8) | 31 (93.9) |  |
| Arrest witnessed by | n=520 | n=487 | n=33 | 0.068^*^ |
| None | 110 (21.2) | 108 (22.2) | 2 (6.1) |  |
| Bystander | 281 (54.0) | 262 (53.8) | 19 (57.6) |  |
| Healthcare provider | 72 (13.8) | 66 (13.6) | 6 (18.2) |  |
| EMS/Private ambulance | 57 (11.0) | 51 (10.5) | 6 (18.2) |  |
| Etiology of OHCA, no. (%) |  |  |  | <0.001^*^ |
| Presumed cardiac etiology | 234 (44.9) | 215 44.1) | 19 (57.6) |  |
| Respiratory | 124 (23.8) | 111 (22.7) | 13 (39.4) |  |
| Drowning | 6 (1.2) | 6 (1.2) | 0 |  |
| Electrocution | 8 (1.5) | 7 (1.4) | 1 (3.0) |  |
| Other | 149 (28.6) | 149 (30.5) | 0 |  |
| Etiology of OHCA |  |  |  | 0.131 |
| Non-cardiac | 287 (55.1) | 273 (55.9) | 14 (42.4) |  |
| Presumed cardiac | 234 (44.9) | 215 (44.1) | 19 (57.6) |  |
| First arrest rhythm, no. (%) |  |  |  | <0.001 |
| VT | 11 (8.1) | 10 (9.3) | 1 (3.7) |  |
| VF | 29 (21.5 | 14 (13.0) | 15 (55.6) |  |
| Unknown shockable rhythm | 53 (39.3 | 52 (48.1) | 1 (3.7) |  |
| Unknown unshockable rhythm | 20 (14.8 | 12 (11.1) | 8 (29.6) |  |
| PEA | 5 (3.7) | 5 (4.6) | 0 |  |
| Asystole | 17 (12.6) | 15 (13.9) | 2 (7.4) |  |
| First arrest rhythm | n=135 | n=108 | n=27 | 0.457 |
| Unshockable rhythm | 42 (31.1) | 32 (29.6) | 10 (37.0) |  |
| Shockable rhythm | 93 (68.9) | 76 (70.4) | 17 (63.0) |  |
| **System-related** |  |  |  |  |
| Types of pre-hospital transportation, no. (%) |  |  |  | <0.001 |
| EMS | 42 (8.1) | 41 (8.4) | 1 (3.0) |  |
| Private ambulance | 223 (42.8) | 197 (40.4) | 26 (78.8) |  |
| Private or public transport | 256 (49.1) | 250 (51.2) | 6 (18.2) |  |
| Resuscitation attempted by EMS/private ambulance, no. (%) | 133/262 (50.8) | 106/235 (45.1) | 27/27 (100.0) | <0.001 |
| Time to initiation of CPR (min), mean (SD) | 7.33/87 (8.70) | 7.99/61 (9.82) | 5.77/26 (5.03) | 0.384^***^ |
| Time to defibrillation at scene (min), mean (SD) | 9.00/36 (6.21) | 7.59/20 (6.95) | 10.76/16 (4.78) | 0.049^***^ |
| **Therapy-related** |  |  |  |  |
| Pharmacotherapy, no. (%) |  |  |  |  |
| Epinephrine (at scene) | 122 (23.4) | 96 (19.7) | 26 (78.8) | <0.001 |
| Epinephrine (at ED) | 480 (92.1) | 448 (91.8) | 32 (97.0) | 0.501^*^ |
| Pre-hospital intervention, no. (%) |  |  |  |  |
| Bystander CPR | 115 (22.1) | 101 (20.7) | 14 (42.4) | 0.004 |
| Pre-hospital defibrillation | 43/135 (31.9) | 26/108 (24.1) | 17/27 (63.0) | <0.001 |
| Bystander AED applied | 14/262 (5.3) | 9/235 (3.8) | 5/27 (18.5 | 0.008^*^ |
| ED defibrillation performed, no. (%) | 68 (13.1) | 63 (12.9) | 5 (15.2) | 0.788^*^ |
| Pre-hospital advanced airway, no. (%) | 108 (20.7) | 81 (16.6) | 27 (81.8) | <0.001 |
| Pre-hospital advanced airway techniques, no. (%) | n=108 | n=81 | n=27 | >0.999^*^ |
| Oral/Nasal ET | 106 (98.1) | 79 (97.5) | 27 (100.0) |  |
| LMA | 1 (0.9) | 1 (1.2) | 0 |  |
| Other | 1 (0.9) | 1 (1.2) | 0 |  |
| Advanced airway used at ED, no. (%) | 297 (57.0) | 291 (59.6) | 6 (18.2) | <0.001 |
| Advanced airway techniques used at ED, no. (%) | n=297 | n=291 | n=6 | >0.999^*^ |
| Oral/Nasal ET | 294 (99.0) | 288 (99.0) | 6 (100.0) |  |
| LMA | 1 (0.3) | 1 (0.3) | 0 |  |
| Other | 2 (0.7) | 2 (0.7) | 0 |  |
| Immediate coronary angiography on admission to hospital, no. (%) |  |  |  |  |
| Emergency PCI performed | 23 (4.4) | 19 (3.9) | 4 (12.1) | 0.050^*^ |
| Emergency CABG performed | 2 (0.4) | 2 (0.4) | 0 | >0.999^*^ |
| Post-resuscitation care, no. (%) |  |  |  |  |
| ECMO therapy initiated | 7/519 (1.3) | 7/486 (1.4) | 0 | >0.999^*^ |
| Hypothermia therapy initiated | 78 (15.0) | 47 (9.6) | 31 (93.9) | <0.001^*^ |
| **Outcomes** |  |  |  |  |
| ROSC, no. (%) |  |  |  |  |
| ROSC at scene/en-route | 98 (18.8) | 71 (14.5) | 27 (81.8) | <0.001 |
| ROSC at ED | 113 (21.7) | 103 (21.1) | 10 (30.3) | 0.215 |
| Outcome of patient at ED, no. (%) |  |  |  | <0.001 |
| Died in ED | 425 (81.6) | 425 (87.1) | 0 |  |
| Admitted | 96 (18.4) | 63 (12.9) | 33 (100.0) |  |
| Patient status, no. (%) |  |  |  | <0.001^*^ |
| Died in the hospital | 41 (7.9) | 41 (8.4) | 0 |  |
| Remains in hospital at 30th day post arrest | 6 (1.2) | 6 (1.2) | 0 |  |
| Discharged alive | 49 (9.4) | 16 (3.3) | 33 (100.0) |  |
| ^a^ The comparison between patients who had post-arrest CPC of 3 to 5 and who had post-arrest CPC of 1 to 2, using the Chi-squared test; ^*^Fisher’s exact test; ^**^Independent-samples T-test; ^***^Mann-Whitney U test. Abbreviations: **AED**, automatic external defibrillation; **CABG**, coronary artery bypass grafting; **CPC**, cerebral performance category; **CPR**, cardiopulmonary resuscitation; **ECMO**, extracorporeal membrane oxygenation; **ED**, emergency department; **EMS**, emergency medical services; **ET**, endotracheal; **LMA**, laryngeal mask airway; **OHCA**, out-of-hospital cardiac arrest; **PCI**, percutaneous coronary intervention; **PEA**, pulseless electrical activity; **ROSC**, return of spontaneous circulation; **SD**, standard deviation; **Time of the day**, period from 8:00 to 20:00 hour; **VF**, ventricular fibrillation; **VT**, ventricular tachycardia. | | | | |

**Table S5**. General characteristics, management, and outcomes of matched cohorts of patients with out-of-hospital cardiac arrest according to the type of bystander cardiopulmonary resuscitation, Vietnam, February 2014 - December 2018

| Characteristics | All cases | No bystander CPR | Bystander CPR | p-value^a^ |
| --- | --- | --- | --- | --- |
| **Hospital participated** | n=212 | n=106 | n=106 |  |
| Hospital |  |  |  | 0.140^*^ |
| Bach Mai, no. (%) | 176 (83.0) | 91 (85.8) | 85 (80.2) |  |
| Hue, no. (%) | 2 (0.9) | 2 (1.9) | 0 |  |
| Cho Ray, no. (%) | 34 (16.0) | 13 (12.3) | 21 (19.8) |  |
| **Patient related** | n=212 | n=106 | n=106 |  |
| Age (year), mean (SD) | 56,56 (17.49) | 60.04 (16.57) | 53.08 (17.78) | 0.004^**^ |
| Age (year), no. (%) |  |  |  | 0.127 |
| < 60 | 121 (57.1) | 55 (51.9) | 66 (62.3) |  |
| ≥ 60 | 91 (42.9) | 51 (48.1) | 40 (37.7) |  |
| Gender, no. (%) |  |  |  | 0.758 |
| Male | 154 (72.6) | 78 (73.6) | 76 (71.7) |  |
| Female | 58 (27.4) | 28 (26.4) | 30 (28.3) |  |
| Past medical history, no. (%) |  |  |  |  |
| Heart disease | 38 (17.9) | 13 (12.3) | 25 (23.6) | 0.032 |
| Diabetes | 30 (14.2) | 12 (11.3) | 18 (17.0) | 0.237 |
| Cancer | 11 (5.2) | 7 (6.6) | 4 (3.8) | 0.353 |
| Hypertension | 47 (22.2) | 21 (19.8) | 26 (24.5) | 0.408 |
| Renal disease | 15 (7.1) | 4 (3.8) | 11 (10.4) | 0.061 |
| Respiratory disease | 37 (17.5) | 15 (14.2) | 22 (20.8) | 0.205 |
| Hyperlipidemia | 0 | 0 | 0 |  |
| Stroke | 6 (2.8) | 5 (4.7) | 1 (0.9) | 0.212^*^ |
| HIV | 0 | 0 | 0 |  |
| Other | 53 (25.0) | 31 (29.2) | 22 (20.8) | 0.153 |
| Past medical history |  |  |  | 0.090 |
| None | 47 (22.2) | 23 (21.7) | 24 (22.6) |  |
| Heart disease | 10 (4.7) | 5 (4.7) | 5 (4.7) |  |
| No heart disease | 127 (59.9) | 70 (66.0) | 57 (53.8) |  |
| Both | 28 (13.2) | 8 (7.5) | 20 (18.9) |  |
| **Event related** | n=212 | n=106 | n=106 |  |
| Location type, no. (%) |  |  |  | 0.002^*^ |
| Home residence | 109 (51.4) | 59 (55.7) | 50 (47.2) |  |
| Healthcare facility | 39 (18.4) | 8 (7.5) | 31 (29.2) |  |
| In EMS/Private ambulance | 40 (18.9) | 24 (22.6) | 16 (15.1) |  |
| Industrial place | 1 (0.5) | 1 (0.9) | 0 |  |
| Nursing home | 1 (0.5) | 0 | 1 (0.9) |  |
| Place of recreation | 2 (0.9) | 1 (0.9) | 1 (0.9) |  |
| Public/Commercial building | 3 (1.4) | 2 (1.9) | 1 (0.9) |  |
| Street/Highway | 6 (2.8) | 3 (2.8) | 3 (2.8) |  |
| Transport center | 1 (0.5) | 1 (0.9) | 0 |  |
| Other | 10 (4.7) | 7 (6.6) | 3 (2.8) |  |
| Location type |  |  |  | <0.001 |
| In EMS/Private ambulance | 40 (18.9) | 24 (22.6) | 16 (15.1) |  |
| Healthcare facility | 40 (18.9) | 8 (7.5) | 32 (30.2) |  |
| Home residence | 109 (51.4) | 59 (55.7) | 50 (47.2) |  |
| Public area | 23 (10.8) | 15 (14.2) | 8 (7.5) |  |
| Time of the day, no. (%) | 64/105 (61.0) | 13/29 (44.8) | 51/76 (67.1) | 0.036 |
| Arrest witnessed by, no. (%) |  |  |  | <0.001 |
| Not witnessed | 84 (39.6) | 82 (77.4) | 2 (1.9) |  |
| Bystander (Lay person) | 11 (5.2) | 6 (5.7) | 5 (4.7) |  |
| Bystander (Family) | 65 (30.7) | 16 (15.1) | 49 (46.2) |  |
| Bystander (Healthcare provider) | 31 (14.6) | 2 (1.9) | 29 (27.4) |  |
| EMS/Private ambulance | 21 (9.9) | 0 | 21 (19.8) |  |
| Witnessed arrest |  |  |  | <0.001 |
| No | 84 (39.6) | 82 (77.4) | 2 (1.9) |  |
| Yes | 128 (60.4) | 24 (22.6) | 104 (98.1) |  |
| Arrest witnessed by |  |  |  | <0.001 |
| None | 84 (39.6) | 82 (77.4) | 2 (1.9) |  |
| Bystander | 42 (19.8) | 8 (7.5) | 34 (32.1) |  |
| Healthcare provider | 65 (30.7) | 16 (15.1) | 49 (46.2) |  |
| EMS/Private ambulance | 21 (9.9) | 0 | 21 (19.8) |  |
| Etiology of OHCA, no. (%) |  |  |  | 0.280^*^ |
| Presumed cardiac etiology | 82 (38.7) | 36 (34.0) | 46 (43.4) |  |
| Respiratory | 65 (30.7) | 38 (35.8) | 27 (25.5) |  |
| Drowning | 4 (1.9) | 1 (0.9) | 3 (2.8) |  |
| Electrocution |  |  |  |  |
| Other | 61 (28.8) | 31 (29.2) | 30 (28.3) |  |
| Etiology of OHCA |  |  |  | 0.158 |
| Non-cardiac | 130 (61.3) | 70 (66.0) | 60 (56.6) |  |
| Presumed cardiac | 82 (38.7) | 36 (34.0) | 46 (43.4) |  |
| First arrest rhythm, no. (%) | n=56 | - | n=56 | - |
| VT | 5 (8.9) | - | 5 (8.9) |  |
| VF | 7 (12.5) | - | 7 (12.5) |  |
| Unknown shockable rhythm | 27 (48.2) | - | 27 (48.2) |  |
| Unknown unshockable rhythm | 7 (12.5) | - | 7 (12.5) |  |
| PEA | 3 (5.4) | - | 3 (5.4) |  |
| Asystole | 7 (12.5) | - | 7 (12.5) |  |
| First arrest rhythm | n=56 | - | n=56 |  |
| Unshockable rhythm | 17 (30.4) | - | 17 (30.4) |  |
| Shockable rhythm | 39 (69.6) | - | 39 (69.6) |  |
| **System related** | n=212 | n=106 | n=106 |  |
| Types of pre-hospital transportation, no. (%) |  |  |  | <0.001 |
| EMS | 16 (7.5) | 1 (0.9) | 15 (14.2) |  |
| Private ambulance | 111 (52.4) | 37 (34.9) | 74 (69.8) |  |
| Private or public transport | 85 (40.1) | 68 (64.2) | 17 (16.0) |  |
| Resuscitation attempted by EMS/private ambulance, no. (%) | 55/125 (44.0) | 0/38 | 55/87 (63.2) | <0.001 |
| Time to initiation of CPR (min), mean (SD) | 5,49 (11.48) | - | 5,49 (11.48) | - |
| Time to defibrillation at scene (min), mean (SD) | 8.50 (8.43) | - | 8.50 (8.43) | - |
| **Therapeutic related** | n=212 | n=106 | n=106 |  |
| Pharmacotherapy, no. (%) |  |  |  |  |
| Epinephrine (at scene) | 52 (24.5) | 0 | 52 (49.1) | <0.001 |
| Epinephrine (at ED) | 196 (92.5) | 99 (93.4) | 97 (91.5) | 0.603 |
| Pre-hospital intervention, no. (%) |  |  |  |  |
| Pre-hospital defibrillation | 12/56 (21.4) | - | 12/56 (21.4) | - |
| Bystander AED applied | 7/125 (5.6) | 0 | 7/87 (8.0) | 0.100^*^ |
| ED defibrillation performed, no. (%) | 24 (11.3) | 6 (5.7) | 18 (17.0) | 0.009 |
| Pre-hospital advanced airway, no. (%) | 43 (20.3) | 0 | 43 (40.6) | <0.001 |
| Pre-hospital advanced airway techniques, no. (%) |  |  |  | - |
| Oral/Nasal ET | - | - | 43 (100.0) |  |
| LMA | - | - | - |  |
| Other | - | - | - |  |
| Advanced airway used at ED, no. (%) | 111 (52.4) | 59 (55.7) | 52 (49.1) | 0.336 |
| Advanced airway techniques used at ED, no. (%) |  |  |  | 0.468^*^ |
| Oral/Nasal ET | 110 (99.1) | 59 (100.0) | 51 (98.1) |  |
| LMA | 0 | 0 | 0 |  |
| Other | 1 (0.9) | 0 | 1 (1.9) |  |
| Immediate coronary angiography on admission to hospital, no. (%) |  |  |  |  |
| Emergency PCI performed | 5 (2.4) | 0 | 5 (4.7) | 0.060 |
| Emergency CABG performed | - | - | - | - |
| Post-resuscitation care, no. (%) |  |  |  |  |
| ECMO therapy initiated | 2 (0.9) | 1 (0.9) | 1 (0.9) | >0.999^*^ |
| Hypothermia therapy initiated | 26 (12.3) | 2 (1.9) | 24 (22.6) | <0.001 |
| **Outcomes** | n=212 | n=106 | n=106 |  |
| ROSC, no. (%) |  |  |  |  |
| ROSC at scene/en-route | 39 (18.4) | 0 | 39 (36.8) | <0.001 |
| ROSC at ED | 49 (23.1) | 18 (17.0) | 31 (29.2) | 0.034 |
| Outcome of patient at ED, no. (%) |  |  |  | <0.001 |
| Died in ED | 185 (87.3) | 106 (100.0) | 79 (74.5) |  |
| Admitted | 27 (12.7) | 0 | 27 (12.7) |  |
| Patient status, no. (%) |  |  |  | <0.001 |
| Died in the hospital | 10 (4.7) | 0 | 10 (9.4) |  |
| Remains in hospital at 30th day post arrest | 0 | 0 | 0 |  |
| Discharged alive | 17 (8.0) | 0 | 17 (16.0) |  |
| Post arrest CPC 1 or 2, n (%) | 14 (6.6) | 0 | 14 (13.2) | <0.001 |
| ^a^ The comparison between patients who did not receive bystander CPR and who received bystander CPR, using the Chi-squared test; ^*^Fisher’s exact test; ^**^Independent-samples T-test; ^***^Mann-Whitney U test. Abbreviations: **AED**, automatic external defibrillation; **CABG**, coronary artery bypass grafting; **CPC**, cerebral performance category; **CPR**, cardiopulmonary resuscitation; **ECMO**, extracorporeal membrane oxygenation; **ED**, emergency department; **EMS**, emergency medical services; **ET**, endotracheal; **LMA**, laryngeal mask airway; **OHCA**, out-of-hospital cardiac arrest; **PCI**, percutaneous coronary intervention; **PEA**, pulseless electrical activity; **ROSC**, return of spontaneous circulation; **SD**, standard deviation; **Time of the day**, period from 8:00 to 20:00 hour; **VF**, ventricular fibrillation; **VT**, ventricular tachycardia. | | | | |

**Table S6**. Factors associated with survival to hospital admission in patients with out-of-hospital cardiac arrest: univariable logistic regression analyses

| Factor | Frequency | OR | 95.0% CI for OR | | p |
| --- | --- | --- | --- | --- | --- |
|  |  |  | Lower | Upper |  |
| **Hospital participated** |  |  |  |  |  |
| Hospital |  |  |  |  |  |
| Bach Mai hospital | 396 | - | - | - | 0.148 |
| Hue hospital | 24 | 2.250 | 0.927 | 5.459 | 0.073 |
| Cho Ray hospital | 101 | 0.847 | 0.469 | 1.531 | 0.583 |
| **Patient-related** |  |  |  |  |  |
| Age (year) |  |  |  |  |  |
| < 60 | 290 | - | - | - | - |
| ≥ 60 | 231 | 0.399 | 0.245 | 0.650 | <0.001 |
| Gender (Male) | 388 | 1.475 | 0.991 | 3.072 | 0.054 |
| Past medical history |  |  |  |  |  |
| None | 107 | - | - | - | <0.001 |
| Heart disease | 24 | 0.086 | 0.011 | 0.661 | 0.018 |
| No heart disease | 278 | 0.322 | 0.190 | 0.544 | <0.001 |
| Both | 61 | 0.483 | 0.229 | 1.020 | 0.056 |
| Past medical history |  |  |  |  |  |
| Heart disease | 85 | 0.746 | 0.393 | 1.418 | 0.372 |
| Diabetes | 64 | 1.125 | 0.582 | 2.173 | 0.726 |
| Cancer | 38 | 0.224 | 0.053 | 0.947 | 0.042 |
| Hypertension | 111 | 0.941 | 0.542 | 1.632 | 0.827 |
| Renal disease | 38 | 0.107 | 0.015 | 0.792 | 0.029 |
| Respiratory disease | 75 | 2.635 | 1.519 | 4.569 | 0.001 |
| Hyperlipidemia | 4 | 4.419 | 0.614 | 31.808 | 0.140 |
| Stroke | 16 | 0.281 | 0.037 | 2.158 | 0.222 |
| HIV | 1 | 0.000 | 0.000 | - | >0.999 |
| Location type |  |  |  |  |  |
| In EMS/Private ambulance | 63 | - | - | - | 0.060 |
| Healthcare facility | 50 | 1.582 | 0.453 | 5.520 | 0.472 |
| Home residence | 358 | 2.971 | 1.150 | 7.675 | 0.025 |
| Public area | 50 | 3.663 | 1.195 | 11.234 | 0.023 |
| Time of the day | 181 | 1.686 | 0.999 | 2.843 | 0.050 |
| Witnessed arrest |  |  |  |  |  |
| No | 110 | - | - | - | - |
| Yes | 410 | 3.484 | 1.634 | 7.430 | 0.001 |
| Arrest witnessed by |  |  |  |  |  |
| None | 110 | - | - | - | 0.011 |
| Bystander | 281 | 3.685 | 1.702 | 7.976 | 0.001 |
| Healthcare provider | 72 | 2.809 | 1.100 | 7.172 | 0.031 |
| EMS/Private ambulance | 57 | 3.400 | 1.301 | 8.888 | 0.013 |
| Etiology of OHCA |  |  |  |  |  |
| Non-cardiac | 287 | - | - | - | - |
| Presumed cardiac | 234 | 1.221 | 0.783 | 1.902 | 0.378 |
| First arrest rhythm |  |  |  |  |  |
| Unshockable rhythm | 42 | - | - | - | - |
| Shockable rhythm | 93 | 0.425 | 0.201 | 0.898 | 0.025 |
| **System-related** |  |  |  |  |  |
| Types of pre-hospital transportation |  |  |  |  |  |
| Ambulance | 324 | - | - | - | - |
| Private or public transport | 197 | 0.293 | 0.168 | 0.512 | <0.001 |
| Resuscitation attempted by EMS/private ambulance | 133 | 10.710 | 5.163 | 22.215 | <0.001 |
| **Therapy-related** |  |  |  |  |  |
| Pharmacotherapy |  |  |  |  |  |
| Epinephrine (at scene) | 122 | 10.400 | 6.332 | 17.082 | <0.001 |
| Epinephrine (at ED) | 480 | 0.667 | 0.320 | 1.432 | 0.307 |
| Prehospital intervention |  |  |  |  |  |
| Bystander CPR | 115 | 1.600 | 0.971 | 2.635 | 0.065 |
| Prehospital defibrillation | 43 | 4.619 | 2.094 | 10.186 | <0.001 |
| Bystander AED applied | 14 | 2.026 | 0.678 | 6.056 | 0.206 |
| Prehospital advanced airway | 108 | 15.024 | 8.948 | 25.227 | <0.001 |
| Intervention at ED |  |  |  |  |  |
| CPR at ED | 16 | 0.287 | 0.037 | 2.200 | 0.230 |
| ED defibrillation performed | 68 | 2.886 | 1.653 | 5.040 | <0.001 |
| Advanced airway used at ED | 297 | 0.319 | 0.201 | 0.508 |  |
| **Outcomes** |  |  |  |  |  |
| ROSC |  |  |  |  |  |
| ROSC at scene/en-route | 98 | 21.273 | 12.335 | 36.686 | <0.001 |
| ROSC at ED | 113 | 4.366 | 2.709 | 7.035 | <0.001 |
| Post arrest CPC 1 or 2 | 33 | 10898044721.217089 | 0 | - | 0.997 |

**Table S7**. Factors associated with survival to hospital discharge in patients with out-of-hospital cardiac arrest: univariable logistic regression analyses

| Factor | Frequency | | OR | | 95.0% CI for OR | | | | P-value |
| --- | --- | --- | --- | --- | --- | --- | --- | --- | --- |
|  |  |  |  |  | Lower | | Upper | |  |
| **Hospital participated** |  | |  | |  | |  | |  |
| Hospital |  | |  | |  | |  | |  |
| Bach Mai hospital | 396 | | - | | - | | - | | 0.034 |
| Hue hospital | 24 | | 0 | | 0 | | - | | 0.998 |
| Cho Ray hospital | 101 | | 0.150 | | 0.036 | | 0.628 | | 0.009 |
| **Patient-related** |  | |  | |  | |  | |  |
| Age (year) |  | |  | |  | |  | |  |
| < 60 | 290 | | - | | - | | - | | - |
| ≥ 60 | 231 | | 0.291 | | 0.142 | | 0.597 | | 0.001 |
| Gender (Male) | 388 | | 2.185 | | 0.957 | | 4.989 | | 0.064 |
| Past medical history |  | |  | |  | |  | |  |
| None | 107 | | - | | - | | - | | <0.001 |
| Heart disease | 24 | | 0 | | 0 | | - | | 0.998 |
| No heart disease | 278 | | 0.242 | | 0.128 | | 0.455 | | <0.001 |
| Both | 61 | | 0.106 | | 0.024 | | 0.462 | | 0.003 |
| Past medical history | |  | |  | |  | |  |  |
| Heart disease | | 85 | | 0.178 | | 0.042 | | 0.746 | 0.018 |
| Diabetes | | 64 | | 0.715 | | 0.272 | | 1.880 | 0.497 |
| Cancer | | 38 | | 0.221 | | 0.030 | | 1.651 | 0.141 |
| Hypertension | | 111 | | 0.724 | | 0.339 | | 1.545 | 0.404 |
| Renal disease | | 38 | | 0.221 | | 0.030 | | 1.651 | 0.141 |
| Respiratory disease | | 75 | | 2.437 | | 1.236 | | 4.805 | 0.010 |
| Hyperlipidemia | | 4 | | 0 | | 0 | | - | 0.999 |
| Stroke | | 16 | | 0 | | 0 | | - | 0.998 |
| HIV | | 1 | | 0 | | 0 | | - | >0.999 |
| **Event-related** |  | |  | |  | |  | |  |
| Location type |  | |  | |  | |  | |  |
| In EMS/Private ambulance | 63 | | - | | - | | - | | 0.407 |
| Healthcare facility | 50 | | 1.277 | | 0.246 | | 6.615 | | 0.771 |
| Home residence | 358 | | 2.305 | | 0.689 | | 7.719 | | 0.176 |
| Public area | 50 | | 2.727 | | 0.647 | | 11.505 | | 0.172 |
| Time of the day | 181 | | 2.065 | | 1.060 | | 4.022 | | 0.033 |
| Witnessed arrest |  | |  | |  | |  | |  |
| No | 110 | | - | | - | | - | | - |
| Yes | 410 | | 4.507 | | 1.374 | | 14.781 | | 0.013 |
| Arrest witnessed by |  | |  | |  | |  | |  |
| None | 110 | | - | | - | | - | | 0.091 |
| Bystander | 281 | | 4.746 | | 1.425 | | 15.812 | | 0.011 |
| Healthcare provider | 72 | | 3.841 | | 0.959 | | 15.378 | | 0.057 |
| EMS/Private ambulance | 57 | | 4.196 | | 1.009 | | 17.455 | | 0.049 |
| Etiology of OHCA |  | |  | |  | |  | |  |
| Non-cardiac | 287 | | - | | - | | - | | - |
| Presumed cardiac | 234 | | 1.435 | | 0.796 | | 2.588 | | 0.230 |
| First arrest rhythm |  | |  | |  | |  | |  |
| Unshockable rhythm | 42 | | - | | - | | - | | - |
| Shockable rhythm | 93 | | 0.591 | | 0.269 | | 1.300 | | 0.191 |
| **System-related** |  | |  | |  | |  | |  |
| Types of pre-hospital transportation |  | |  | |  | |  | |  |
| Ambulance | 324 | | - | | - | | - | | - |
| Private or public transport | 197 | | 0.391 | | 0.190 | | 0.802 | | 0.010 |
| Resuscitation attempted by EMS/private ambulance | 133 | | 51.200 | | 6.907 | | 379.550 | | <0.001 |
| **Therapy-related** |  | |  | |  | |  | |  |
| Pharmacotherapy |  | |  | |  | |  | |  |
| Epinephrine (at scene) | 122 | | 14.038 | | 7.026 | | 28.048 | | <0.001 |
| Epinephrine (at ED) | 480 | | 4.444 | | 0.598 | | 33.057 | | 0.145 |
| Prehospital intervention |  | |  | |  | |  | |  |
| Bystander CPR | 115 | | 2.027 | | 1.081 | | 3.802 | | 0.028 |
| Prehospital defibrillation | 43 | | 4.211 | | 1.898 | | 9.341 | | <0.001 |
| Bystander AED applied | 14 | | 3.497 | | 1.105 | | 11.060 | | 0.033 |
| Prehospital advanced airway | 108 | | 17.414 | | 8.662 | | 35.009 | | <0.001 |
| Intervention at ED |  | |  | |  | |  | |  |
| CPR at ED | 16 | | 0.633 | | 0.082 | | 4.900 | | 0.662 |
| ED defibrillation performed | 68 | | 1.575 | | 0.727 | | 3.411 | | 0.249 |
| Advanced airway used at ED | 297 | | 0.213 | | 0.108 | | 0.419 | | <0.001 |
| **Outcomes** |  | |  | |  | |  | |  |
| ROSC |  | |  | |  | |  | |  |
| ROSC at scene/en-route | 98 | | 23.721 | | 11.504 | | 48.912 | | <0.001 |
| ROSC at ED | 113 | | 2.304 | | 1.236 | | 4.296 | | 0.009 |
| Post arrest CPC 1 or 2 | 33 | | 47656508499.422680 | | 0 | | - | | 0.997 |

**Table S8**. Factors associated with good neurological survival at hospital discharge in patients with out-of-hospital cardiac arrest: univariable logistic regression analyses

| Factor | Frequency | OR | 95.0% CI for OR | | p-value |
| --- | --- | --- | --- | --- | --- |
|  |  |  | Lower | Upper |  |
| **Hospital participated** |  |  |  |  |  |
| Hospital |  |  |  |  |  |
| Bach Mai hospital | 396 | - | - | - | 0.151 |
| Hue hospital | 24 | 0 | 0 | - | 0.998 |
| Cho Ray hospital | 101 | 0.238 | 0.056 | 1.011 | 0.052 |
| **Patient-related** |  |  |  |  |  |
| Age (year) |  |  |  |  |  |
| < 60 | 290 | - | - | - | - |
| ≥ 60 | 231 | 0.260 | 0.105 | 0.640 | 0.003 |
| Gender (Male) | 388 | 1.991 | 0.753 | 5.267 | 0.165 |
| Past medical history |  |  |  |  |  |
| None | 107 | - | - | - | <0.001 |
| Heart disease | 24 | 0 | 0 | - | 0.998 |
| No heart disease | 278 | 0.227 | 0.108 | 0.479 | <0.001 |
| Both | 61 | 0.077 | 0.010 | 0.592 | 0.014 |
| Past medical history |  |  |  |  |  |
| Heart disease | 85 | 0.131 | 0.018 | 0.975 | 0.047 |
| Diabetes | 64 | 0.616 | 0.182 | 2.082 | 0.436 |
| Cancer | 38 | 0.338 | 0.045 | 2.543 | 0.292 |
| Hypertension | 111 | 0.558 | 0.210 | 1.480 | 0.558 |
| Renal disease | 38 | 0.000 | 0.000 | - | 0.998 |
| Respiratory disease | 75 | 2.914 | 1.348 | 6.299 | 0.007 |
| Hyperlipidemia | 4 | 0 | 0 | - | 0.999 |
| Stroke | 16 | 0 | 0 | - | 0.999 |
| HIV | 1 | 0 | 0 | - | >0.999 |
| **Event-related** |  |  |  |  |  |
| Location type |  |  |  |  |  |
| In EMS/Private ambulance | 63 | - | - | - | 0.948 |
| Healthcare facility | 50 | 1.277 | 0.246 | 6.615 | 0.771 |
| Home residence | 358 | 1.437 | 0.420 | 4.923 | 0.564 |
| Public area | 50 | 1.277 | 0.246 | 6.615 | 0.771 |
| Time of the day | 181 | 2.624 | 1.145 | 6.014 | 0.023 |
| Witnessed arrest |  |  |  |  |  |
| No | 110 | - | - | - |  |
| Yes | 410 | 4.417 | 1.040 | 18.751 | 0.044 |
| Arrest witnessed by |  |  |  |  |  |
| None | 110 | - | - | - | 0.161 |
| Bystander | 281 | 3.916 | 0.897 | 17.103 | 0.070 |
| Healthcare provider | 72 | 4.909 | 0.962 | 25.039 | 0.056 |
| EMS/Private ambulance | 57 | 6.353 | 1.239 | 32.574 | 0.027 |
| Etiology of OHCA |  |  |  |  |  |
| Non-cardiac | 287 | - | - | - | - |
| Presumed cardiac | 234 | 1.723 | 0.845 | 3.516 | 0.135 |
| First arrest rhythm |  |  |  |  |  |
| Unshockable rhythm | 42 | - | - | - | - |
| Shockable rhythm | 93 | 0.716 | 0.296 | 1.732 | 0.458 |
| **System-related** |  |  |  |  |  |
| Types of pre-hospital transportation |  |  |  |  |  |
| Ambulance | 324 | - | - | - | - |
| Private or public transport | 197 | 0.346 | 0.140 | 0.853 | 0.021 |
| Resuscitation attempted by EMS/private ambulance | 133 | 411488932.053261 | 0 | - | 0.996 |
| **Therapy-related** |  |  |  |  |  |
| Pharmacotherapy |  |  |  |  |  |
| Epinephrine (at scene) | 122 | 0.066 | 0.028 | 0.156 | <0.001 |
| Epinephrine (at ED) | 480 | 0.350 | 0.047 | 2.629 | 0.308 |
| Prehospital intervention |  |  |  |  |  |
| Bystander CPR | 115 | 2.823 | 1.368 | 5.825 | 0.005 |
| Prehospital defibrillation | 43 | 5.362 | 2.186 | 13.148 | <0.001 |
| Bystander AED applied | 14 | 5.707 | 1.758 | 18.528 | 0.004 |
| Prehospital advanced airway | 108 | 22.611 | 9.045 | 56.521 | <0.001 |
| Intervention at ED |  |  |  |  |  |
| CPR at ED | 16 | 0 | 0 | - | 0.999 |
| ED defibrillation performed | 68 | 1.205 | 0.449 | 3.234 | 0.712 |
| Advanced airway used at ED | 297 | 0.150 | 0.061 | 0.371 | <0.001 |
| **Outcomes** |  |  |  |  |  |
| ROSC |  |  |  |  |  |
| ROSC at scene/en-route | 98 | 26.430 | 10.536 | 66.299 | <0.001 |
| ROSC at ED | 113 | 1.625 | 0.750 | 3.523 | 0.219 |

**Table S9**. Factors associated with survival to hospital admission in patients with out-of-hospital cardiac arrest: multivariable logistic regression analysis (backward elimination)

| Steps | Factor | Unit | OR | 95.0% CI for OR | | p-value |
| --- | --- | --- | --- | --- | --- | --- |
|  |  |  |  | Lower | Upper |  |
| 1 | Age (year) |  |  |  |  |  |
|  | < 60 | % | - | - | - | - |
|  | ≥ 60 | % | 0.543 | 0.304 | 0.967 | 0.038 |
|  | Past medical history |  |  |  |  |  |
|  | Heart disease | % | 0.688 | 0.311 | 1.520 | 0.355 |
|  | Cancer | % | 0.211 | 0.046 | 0.969 | 0.045 |
|  | Renal disease | % | 0.062 | 0.008 | 0.484 | 0.008 |
|  | Respiratory disease | % | 3.135 | 1.563 | 6.289 | 0.001 |
|  | Location type |  |  |  |  |  |
|  | In EMS/Private ambulance | % | - | - | - | 0.004 |
|  | Healthcare facility | % | 2.949 | 0.608 | 14.303 | 0.179 |
|  | Home residence | % | 7.747 | 2.254 | 28.619 | 0.001 |
|  | Public area | % | 10.306 | 2.359 | 45.024 | 0.002 |
|  | Witnessed arrest |  |  |  |  |  |
|  | No | % | - | - | - | - |
|  | Yes | % | 3.450 | 1.364 | 8.728 | 0.009 |
|  | Etiology of OHCA |  |  |  |  |  |
|  | Non-cardiac |  | - | - | - | - |
|  | Presumed cardiac |  | 1.694 | 0.916 | 3.133 | 0.093 |
|  | Types of pre-hospital transportation |  |  |  |  |  |
|  | Ambulance | % | - | - | - | - |
|  | Private or public transport | % | 0.212 | 0.108 | 0.414 | <0.001 |
|  | Bystander CPR |  |  |  |  |  |
|  | No | % | - | - | - | - |
|  | Yes | % | 1.137 | 0.593 | 2.183 | 0.699 |
|  | Constant |  | 0.919 | - | - | <0.001 |
| 2 | Age (year) |  |  |  |  |  |
|  | < 60 | % | - | - | - | - |
|  | ≥ 60 | % | 0.537 | 0.302 | 0.955 | 0.034 |
|  | Past medical history |  |  |  |  |  |
|  | Heart disease | % | 0.700 | 0.319 | 1.536 | 0.374 |
|  | Cancer | % | 0.202 | 0.045 | 0.916 | 0.038 |
|  | Renal disease | % | 0.062 | 0.008 | 0.478 | 0.008 |
|  | Respiratory disease | % | 3.128 | 1.561 | 6.268 | 0.001 |
|  | Location type |  |  |  |  |  |
|  | In EMS/Private ambulance | % | - | - | - | 0.004 |
|  | Healthcare facility | % | 3.148 | 0.672 | 14.755 | 0.146 |
|  | Home residence | % | 7.673 | 2.237 | 26.319 | 0.001 |
|  | Public area | % | 10.335 | 2.368 | 45.110 | 0.002 |
|  | Witnessed arrest |  |  |  |  |  |
|  | No | % | - | - | - | - |
|  | Yes | % | 3.536 | 1.411 | 8.863 | 0.007 |
|  | Etiology of OHCA |  |  |  |  |  |
|  | Non-cardiac |  | - | - | - | - |
|  | Presumed cardiac |  | 1.665 | .907 | 3.056 | 0.100 |
|  | Types of pre-hospital transportation |  |  |  |  |  |
|  | Ambulance | % | - | - | - | - |
|  | Private or public transport | % | 0.206 | 0.107 | 0.397 | <0.001 |
|  | Constant |  | 0.020 | - | - | <0.001 |
| 3 | Age (year) |  |  |  |  |  |
|  | < 60 | % | - | - | - | - |
|  | ≥ 60 | % | 0.514 | 0.291 | 0.907 | 0.022 |
|  | Past medical history |  |  |  |  |  |
|  | Cancer | % | 0.199 | 0.044 | 0.901 | 0.036 |
|  | Renal disease | % | 0.058 | 0.007 | 0.446 | 0.006 |
|  | Respiratory disease | % | 2.959 | 1.495 | 5.858 | 0.002 |
|  | Location type |  |  |  |  |  |
|  | In EMS/Private ambulance | % | - | - | - | 0.003 |
|  | Healthcare facility | % | 3.184 | 0.679 | 14.925 | 0.142 |
|  | Home residence | % | 7.786 | 2.276 | 26.639 | 0.001 |
|  | Public area | % | 10.736 | 2.467 | 46.732 | 0.002 |
|  | Witnessed arrest |  |  |  |  |  |
|  | No | % | - | - | - | - |
|  | Yes | % | 3.450 | 1.380 | 8.622 | 0.008 |
|  | Etiology of OHCA |  |  |  |  |  |
|  | Non-cardiac |  | - | - | - | - |
|  | Presumed cardiac |  | 1.521 | .857 | 2.699 | 0.152 |
|  | Types of pre-hospital transportation |  |  |  |  |  |
|  | Ambulance | % | - | - | - | - |
|  | Private or public transport | % | 0.205 | 0.106 | 0.395 | <0.001 |
|  | Constant |  | 0.020 |  |  | <0.001 |
| 4 | Age (year) |  |  |  |  |  |
|  | < 60 | % | - | - | - | - |
|  | ≥ 60 | % | 0.545 | 0.311 | 0.955 | 0.034 |
|  | Past medical history |  |  |  |  |  |
|  | Cancer | % | 0.167 | 0.038 | 0.740 | 0.018 |
|  | Renal disease | % | 0.059 | 0.008 | 0.453 | 0.006 |
|  | Respiratory disease | % | 2.490 | 1.320 | 4.697 | 0.005 |
|  | Location type |  |  |  |  |  |
|  | In EMS/Private ambulance | % | - | - | - | 0.003 |
|  | Healthcare facility | % | 3.175 | 0.679 | 14.848 | 0.142 |
|  | Home residence | % | 7.827 | 2.294 | 26.708 | 0.001 |
|  | Public area | % | 10.330 | 2.384 | 44.757 | 0.002 |
|  | Witnessed arrest |  |  |  |  |  |
|  | No | % | - | - | - | - |
|  | Yes | % | 3.657 | 1.471 | 9.091 | 0.005 |
|  | Types of pre-hospital transportation |  |  |  |  |  |
|  | Ambulance | % | - | - | - | - |
|  | Private or public transport | % | 0.204 | 0.106 | 0.392 | <0.001 |
|  | Constant |  | 0.024 | - | - | <0.001 |

**Table S10** Factors associated with survival to hospital discharge in patients with out-of-hospital cardiac arrest: multivariable logistic regression analysis (backward elimination)

| Steps | Factor | Unit | OR | 95.0% CI for OR | | p-value |
| --- | --- | --- | --- | --- | --- | --- |
|  |  |  |  | Lower | Upper |  |
| 1 | Age (year) |  |  |  |  |  |
|  | < 60 | % | - | - | - | - |
|  | ≥ 60 | % | 0.355 | 0.166 | 0.758 | 0.007 |
|  | Past medical history |  |  |  |  |  |
|  | Heart disease | % | 0.076 | 0.016 | 0.373 | 0.001 |
|  | Respiratory disease | % | 4.278 | 1.851 | 9.888 | 0.001 |
|  | Witnessed arrest |  |  |  |  |  |
|  | No | % | - | - | - | - |
|  | Yes | % | 3.162 | 0.912 | 10.970 | 0.070 |
|  | Etiology of OHCA |  |  |  |  |  |
|  | Non-cardiac |  | - | - | - | - |
|  | Presumed cardiac |  | 3.231 | 1.518 | 6.876 | 0.002 |
|  | Types of pre-hospital transportation |  |  |  |  |  |
|  | Ambulance | % | - | - | - | - |
|  | Private or public transport | % | 0.543 | 0.242 | 1.218 | 0.138 |
|  | Bystander CPR |  |  |  |  |  |
|  | No | % | - | - | - | - |
|  | Yes | % | 1.732 | 0.851 | 3.523 | 0.130 |
|  | Constant |  | 0.032 | - | - | <0.001 |
| 2 | Age (year) |  |  |  |  |  |
|  | < 60 | % | - | - | - | - |
|  | ≥ 60 | % | 0.329 | 0.155 | 0.698 | 0.004 |
|  | Past medical history |  |  |  |  |  |
|  | Heart disease | % | 0.073 | 0.015 | 0.356 | 0.001 |
|  | Respiratory disease | % | 4.310 | 1.869 | 9.941 | 0.001 |
|  | Witnessed arrest |  |  |  |  |  |
|  | No | % | - | - | - | - |
|  | Yes | % | 3.625 | 1.057 | 12.431 | 0.041 |
|  | Etiology of OHCA |  |  |  |  |  |
|  | Non-cardiac |  | - | - | - | - |
|  | Presumed cardiac |  | 3.337 | 1.570 | 7.094 | 0.002 |
|  | Bystander CPR |  |  |  |  |  |
|  | No | % | - | - | - | - |
|  | Yes | % | 1.962 | 0.980 | 3.929 | 0.057 |
|  | Constant |  | 0.023 | - | - | <0.001 |

**Table S11**. Factors associated with good neurological status in patients with out-of-hospital cardiac arrest: multivariable logistic regression analysis (backward elimination)

| Steps | Factor | Unit | OR | 95.0% CI for OR | | p-value |
| --- | --- | --- | --- | --- | --- | --- |
|  |  |  |  | Lower | Upper |  |
| 1 | Age (year) |  |  |  |  |  |
|  | < 60 | % | - | - | - | - |
|  | ≥ 60 | % | 0.288 | 0.111 | 0.748 | 0.011 |
|  | Past medical history |  |  |  |  |  |
|  | Heart disease | % | 0.029 | 0.003 | 0.276 | 0.002 |
|  | Respiratory disease | % | 8.105 | 2.741 | 23.968 | <0.001 |
|  | Witnessed arrest |  |  |  |  |  |
|  | No | % | - | - | - | - |
|  | Yes | % | 2.737 | 0.569 | 12.567 | 0.195 |
|  | Etiology of OHCA |  |  |  |  |  |
|  | Non-cardiac |  | - | - | - | - |
|  | Presumed cardiac |  | 6.513 | 2.347 | 18.072 | <0.001 |
|  | Types of pre-hospital transportation |  |  |  |  |  |
|  | Ambulance | % | - | - | - | - |
|  | Private or public transport | % | 0.632 | 0.236 | 1.692 | 0.361 |
|  | Bystander CPR |  |  |  |  |  |
|  | No | % | - | - | - | - |
|  | Yes | % | 2.810 | 1.221 | 6.468 | 0.015 |
|  | Constant |  | 0.012 | - | - | <0.001 |
| 2 | Age (year) |  |  |  |  |  |
|  | < 60 | % | - | - | - | - |
|  | ≥ 60 | % | 0.273 | 0.106 | 0.703 | 0.007 |
|  | Past medical history |  |  |  |  |  |
|  | Heart disease | % | 0.027 | 0.003 | 0.260 | 0.002 |
|  | Respiratory disease | % | 8.280 | 2.795 | 24.532 | <0.001 |
|  | Witnessed arrest |  |  |  |  |  |
|  | No | % | - | - | - | - |
|  | Yes | % | 3.052 | 0.676 | 13.780 | 0.147 |
|  | Etiology of OHCA |  |  |  |  |  |
|  | Non-cardiac |  | - | - | - | - |
|  | Presumed cardiac |  | 6.724 | 2.420 | 18.681 | <0.001 |
|  | Bystander CPR |  |  |  |  |  |
|  | No | % | - | - | - | - |
|  | Yes | % | 3.089 | 1.369 | 6.970 | 0.007 |
|  | Constant |  | 0.009 | - | - | <0.001 |
| 3 | Age (year) |  |  |  |  |  |
|  | < 60 | % | - | - | - | - |
|  | ≥ 60 | % | 0.273 | 0.106 | 0.702 | 0.007 |
|  | Past medical history |  |  |  |  |  |
|  | Heart disease | % | 0.027 | 0.003 | 0.265 | 0.002 |
|  | Respiratory disease | % | 8.386 | 2.834 | 24.812 | <0.001 |
|  | Etiology of OHCA |  |  |  |  |  |
|  | Non-cardiac |  | - | - | - | - |
|  | Presumed cardiac |  | 7.236 | 2.611 | 20.053 | <0.001 |
|  | Bystander CPR |  |  |  |  |  |
|  | No | % | - | - | - | - |
|  | Yes | % | 3.624 | 1.629 | 8.063 | 0.002 |
|  | Constant |  | 0.022 | - | - | <0.001 |

**Table S12**. Breakdown of missing data

| Characteristics | All cases  (n=521) |
| --- | --- |
| **Hospital participated** |  |
| Hospital | 0 |
| **Patient-related** |  |
| Age (year) | 0 |
| Gender | 0 |
| Past medical history |  |
| Heart disease | 51 |
| Diabetes | 51 |
| Cancer | 51 |
| Hypertension | 51 |
| Renal disease | 51 |
| Respiratory disease | 51 |
| Hyperlipidemia | 51 |
| Stroke | 51 |
| HIV | 51 |
| Other | 51 |
| Past medical history | 51 |
| **Event-related** |  |
| Location type, | 0 |
| Time of the day, | 201 |
| Arrest witnessed by, | 1 |
| Etiology of OHCA, | 0 |
| First arrest rhythm, | 386 |
| **System-related** |  |
| Types of pre-hospital transportation, | 0 |
| Resuscitation attempted by EMS/private ambulance, | 259 |
| Time to initiation of CPR (min), mean (SD) | 434 |
| Time to defibrillation at scene (min), mean (SD) | 435 |
| **Therapy-related** |  |
| Pharmacotherapy, |  |
| Epinephrine (at scene) | 0 |
| Epinephrine (at ED) | 0 |
| Pre-hospital intervention, |  |
| Pre-hospital defibrillation | 386 |
| Bystander AED applied | 259 |
| ED defibrillation performed, | 0 |
| Pre-hospital advanced airway, | 0 |
| Advanced airway used at ED, | 0 |
| Immediate coronary angiography on admission to hospital, |  |
| Emergency PCI performed | 0 |
| Emergency CABG performed | 0 |
| Post-resuscitation care, |  |
| ECMO therapy initiated | 2 |
| Hypothermia therapy initiated | 0 |
| **Outcomes** |  |
| ROSC, |  |
| ROSC at scene/en-route | 0 |
| ROSC at ED | 0 |
| Outcome of patient at ED | 0 |
| Patient status | 0 |
| Post arrest CPC 1 or 2, n (%) | 0 |
